# Effects of the Yeast RNA-Binding Protein Whi3 on the Half-Life and Abundance of *CLN3* mRNA and Other Targets

**DOI:** 10.1371/journal.pone.0084630

**Published:** 2013-12-30

**Authors:** Ying Cai, Bruce Futcher

**Affiliations:** Department of Molecular Genetics and Microbiology, Stony Brook University, Stony Brook, New York, United States of America; Dartmouth College, United States of America

## Abstract

Whi3 is an RNA binding protein known to bind the mRNA of the yeast G1 cyclin gene *CLN3*. It inhibits *CLN3* function, but the mechanism of this inhibition is unclear; in previous studies, Whi3 made no observable difference to *CLN3* mRNA levels, translation, or protein abundance. Here, we re-approach this issue using microarrays, RNA-Seq, ribosome profiling, and other methods. By multiple methods, we find that the *whi3* mutation causes a small but consistent increase in the abundance of hundreds of mRNAs, including the *CLN3* mRNA. The effect on various mRNAs is roughly in proportion to the density of GCAU or UGCAU motifs carried by these mRNAs, which may be a binding site for Whi3. mRNA instability of Whi3 targets may in part depend on a 3′ AU rich element (ARE), AUUUUA. In addition, the *whi3* mutation causes a small increase in the translational efficiency of *CLN3* mRNA. The increase in *CLN3* mRNA half-life and abundance together with the increase in translational efficiency is fully sufficient to explain the small-cell phenotype of *whi3* mutants. Under stress conditions, Whi3 becomes a component of P-bodies or stress granules, but Whi3 also acts under non-stress condition, when no P-bodies are visible. We suggest that Whi3 may be a very broadly-acting, but mild, modulator of mRNA stability. In *CLN3*, Whi3 may bind to the 3′ GCAU motifs to attract the Ccr4-Not complex to promote RNA deadenylation and turnover, and Whi3 may bind to the 5′ GCAU motifs to inhibit translation.

## Introduction

*WHI3* was discovered in a screen for mutants that commit to cell division at an unusually small cell size (“Wee” or “Whi” mutants) [1]. By sequence, it is an RNA binding protein of the RRM type, and biochemical studies showed that it binds to the mRNA of *CLN3* [2], which encodes a G1 cyclin, and which also affects cell size [3]. Binding of Whi3 to *CLN3* mRNA depends on repeats of the motif GCAU in the *CLN3* mRNA [4]. Just two-fold over-expression of *CLN3* results in exactly the same small-cell phenotype as the *whi3* mutant [1], [3]. Thus, it was suggested that Whi3 might be an inhibitor of *CLN3*, and that in a *whi3* mutant, *CLN3* would be about two-fold hyperactive, allowing commitment to division at small cell size [1], [2]. Indeed, while a *whi3* single mutant has small cells, a *whi3 cln3* double mutant has large cells, exactly like a *cln3* single mutant [2]. The genetic result that *whi3* has no effect on size in a *cln3* mutant strongly supports the idea that the *whi3* cell size phenotype is due to inhibition of *CLN3* at some level.

However, it has been difficult to understand exactly how Whi3 inhibits *CLN3*. Northern analysis failed to show any difference in *CLN3* mRNA levels between wild-type cells and *whi3* mutants; Western analysis failed to show any difference in Cln3 protein levels; and polysome profiling failed to show any difference in the translation of Cln3 [2].

Another kind of idea is that Whi3 forms aggregates or foci [5], [6], and that translation of bound *CLN3* therefore occurs close to these foci, raising the local concentration of Cln3 protein. Wang et al. showed that Whi3 binds Cdc28 protein as well as *CLN3* mRNA. In principle, Whi3 could therefore bind the complex between Cdc28 and the locally-translated Cln3, thus functioning as a cytoplasmic retention device for Cln3-Cdc28 complexes [5]. Since Cln3-Cdc28 acts in the nucleus, this cytoplasmic retention would inhibit Cln3 function. However, there are difficulties with this hypothesis. First, despite several genome-wide screens for protein-protein interactions, and despite multiple screens for protein-protein interactions involving Cdc28 or its homologs in other organisms, other workers have not seen physical interactions between Cdc28 and Whi3. Second, although Aldea and co-workers have shown cytoplasmic Cln3 (the Cln3 presumably retained in the cytoplasm by Whi3) [5], [7], other workers have seen only nuclear Cln3 [8], [9] (Zhao and Futcher, unpublished). Cln3 is a non-abundant protein, and accurate localization is difficult.

Third and most important, the *whi3* mutant has many other phenotypes beyond small cell size. Some of these (poor sporulation; a lack of invasive growth; a lack of pseudohyphal growth) are probably also due to the inhibition of *CLN3*, since a *cln3* mutant is epistatic to *whi3* for these phenotypes [2]. On the other hand, many other phenotypes of *whi3* have no apparent connection to *CLN3*. These phenotypes include sensitivity to oleate, Calcofluor white, and Congo Red, which cannot easily be explained by deregulation of Cln3 activity [4], [10]. In addition, Colomina et al. have shown that Whi3 binds a large number of different mRNAs, suggesting it has targets beyond *CLN3* [4]. The idea that Whi3 binds Cdc28 and so is a cytoplasmic retention device for Cln3-Cdc28 complexes could explain its inhibition of Cln3, but it seems difficult to generalize this model to dozens or hundreds of other targets. Therefore, with the recent availability of methods of higher accuracy and precision, we have re-investigated the effects of Whi3 on *CLN3* mRNA and translation.

Whi3 has a homolog called Whi4 [1]. The *whi4* mutant has little phenotype on its own, but the *whi3 whi4* mutant has even smaller cells than the *whi3* mutant. In addition, the *whi3 whi4* mutant is somewhat sick and slow growing. Because the *whi3* single mutant has obvious phenotypes, and yet is not sick, we have mainly investigated the single mutant. In selected situations, we have also studied the *whi3 whi4* double mutant.

Whi3 is reasonably well conserved among fungi. There is high conservation in and around the RNA binding domain. This region includes a putative site for phosphorylation by cyclic AMP dependent protein kinase [11], [12]; this site could regulate Whi3 function. In addition, Whi3 and Whi4 have a striking Q-rich domain, and a Q-rich domain is a prominent feature of many of the homologs. In some homologs the Q-rich domain has expanded dramatically (e.g., the homologs of *S. bayanus*, *A. gossypii*), while in other cases the Q-rich domain is reduced or perhaps absent (e.g., in *scw1* of *S. pombe*). The Q-rich domain is involved in the formation of Whi3 aggregates in *Ashbya gossypii*, where the Q-rich domain is particularly large [6].

## Results

### Identification of mRNAs Bound by Whi3

Colomina et al. [4] immunoprecipitated Whi3 and obtained a list of 326 co-precipitating mRNAs. To confirm and extend these results, we likewise used RNA immunoprecipitation followed by microarray analysis (RIP-chip) to find RNA targets bound by Whi3. An epitope-tagged Whi3 protein (Whi3-TAP) expressed from its endogenous promoter was immunoprecipitated. As a control, the same experiment was performed with a mutant Whi3 protein lacking its RNA binding domain (the RRM). Multiple experiments were done with each of the wild-type and RRM-deleted Whi3 strains, and the 262 mRNAs whose net enrichment was at least 1.65 standard deviations above the mean (see Materials and Methods) were defined as putative targets of Whi3. *CLN3* mRNA was one of these mRNAs, and ranked as the 70th most-enriched RNA. Our list of 262 mRNAs had 111 mRNAs over-lapping with the previous list of 326 mRNAs [4] (for lists, see Supplementary Tables S1 and S2); this overlap had a p-value of about 10^−74^. The large overlap and small p-value suggest that the two independent studies are substantially in agreement, and are likely identifying real targets of Whi3.

Table 1 shows all 21 mRNAs from the joint list of 111 that are enriched four-fold or more in the immunoprecipitate, and 19 additional mRNAs of interest. Some of these genes are important for cell cycle (the G1 cyclins *CLN3* and *PCL2*; *CDC6*, *NDD1*, *NRM1*), for protein synthesis (the eIF5A elongation factor homologs *HYP2* and *ANB1*, and *SNU13*, *RPA14*, *TEF4*), for cell wall synthesis and maintenance; for lipid metabolism; and for transport of small molecules. The presence of *OLE1* (oleic acid requiring) on the list could be connected to the oleic acid sensitivity of *whi3* [10]. Analysis of our 262 mRNA targets reveals a statistically-significant enrichment of mRNAs encoding proteins associated with membranes, particularly transporters; 91 out of 262 entries (34.7%, p-value = 4.3×10^−5^) are annotated as membrane associated, showing a moderate enrichment compared to the background frequency of 21.3%. Among these 91 RNAs, 29 encode for proteins having transmembrane transporter activity (31.9%, p = 0.007). Similarly, the previous study of Colomina et al. found an enrichment of proteins involved with the cell wall, ER, or plasma membrane, and found transporters. Colomina et al. studied the cell wall defects of *whi3* mutants, showing that *whi3* mutants were sensitive to Calcofluor White and Congo Red [4]. However, while the enrichments of genes in these various functional categories are statistically significant, they are also modest, and many of the target mRNAs do not belong to any of these categories. Overall, it appears that Whi3 interacts with a broad variety of mRNAs, with no single strong, functional theme obvious to us.

**Table 1:** Partial joint list of Whi3 targets.

Common Name	SysName	IP Enrichment^1^	rank^2^	Function
SNU13	YEL026W	3.8	1	RNA binding protein; rRNA processing, mRNA splicing
YGR146C	YGR146C	3.0	2	Unknown
YMR122W-A	YMR122W-A	2.8	3	Unknown
FUR1	YHR128W	2.6	4	Uracil phosphoribosyltransferase
MBF1	YOR298C-A	2.6	5	Transcriptional coactivator, bridges Gcn4 and Spt15
KAP95	YLR347C	2.5	6	Karyopherin beta, nuclear import
YGR035C	YGR035C	2.4	7	Unknown
ANB1	YJR047C	2.4	8	Translation elongation factor eIF-5A
YOR052C	YOR052C	2.3	9	Unknown
SML1	YML058W	2.3	10	Ribonucleotide reductase inhibitor
OLE1	YGL055W	2.2	11	Delta(9) fatty acid desaturase
HYP2	YEL034W	2.1	12	Translation initiation factor eIF-5A,
YEL033W	YEL033W	2.1	13	Unknown
SAM1	YLR180W	2.1	14	S-adenosylmethionine synthetase
RPA14	YDR156W	2.1	15	RNA polymerase I subunit A14
PHO84	YML123C	2.0	16	High-affinity inorganic phosphate (Pi) transporter
TEF4	YKL081W	2.0	17	Translation elongation factor EF-1 gamma
YKR075C	YKR075C	2.0	18	Unknown
MRH1	YDR033W	2.0	19	Unknown
MDM32	YOR147W	2.0	20	Mitochondrial inner membrane
NDD1	YOR372C	2.0	21	M-phase transcriptional activator
BSC1	YDL037C	1.9	22	Cell surface flocculin
GPI18	YBR004C	1.8	23	mannosyltransferase
IPT1	YDR072C	1.7	26	Inositolphosphotransferase, sphingolipid synthesis
GPA2	YER020W	1.7	27	G protein; glucose signalling
FEN1	YCR034W	1.7	29	Fatty acid elongase, sphingolipid synthesis
HXT1	YHR094C	1.7	31	Low-affinity glucose transporter
LCB1	YMR296C	1.6	32	serine palmitoyltransferase, sphingolipid synthesis
NSG2	YNL156C	1.5	36	regulation of sterol synthesis
CDC6	YJL194W	1.5	37	Initiation of DNA replication
DHH1	YDL160C	1.4	42	DExD/H-box helicase, mRNA decapping and decay
UTR2	YEL040W	1.4	43	Cell wall protein, transfer of chitin
OST3	YOR085W	1.3	44	oligosaccharyltransferase complex
CLN3	YAL040C	1.3	47	G1 cyclin, activates Start
NRM1	YNR009W	1.3	51	co-repressor of MBF transcription factor
KRE1	YNL322C	1.3	53	Cell wall, beta-glucan assembly
YEA4	YEL004W	1.3	57	required for chitin synthesis
MUP1	YGR055W	1.2	67	High affinity methionine permease
MUP3	YHL036W	1.1	70	Low affinity methionine permease
PCL2	YDL127W	0.9	100	G1 cyclin

^1^ IP Enrichment is log2 of the enrichment in the RIP-chip (i.e., log2(Whi3-tagged/rrm whi3-tagged)).

^2^ rank is the rank of genes in the joint list.

### RNA Sequence Motifs Associated with Whi3 Targets

Previous studies found that the mRNA targets of Whi3 are enriched with the motif GCAU [4], [13] or UGCAU [14]. The algorithm FIRE [15], which uses mutual information including positional information, has some advantages over previously-used motif search algorithms. Application of discontinuous FIRE to our dataset identified two motifs that are enriched in the 3′ UTRs of Whi3 targets. The first and most significant was, as previously, UGCAU, with a Z-score of 25.9 (i.e., 26 standard deviations from expectation). Despite this, the actual enrichment is modest; the Whi3 RIP-chip targets have on average 3.6 GCAU motifs per kb, while non-targets have 3.1. It is possible that the GCAU motifs are somewhat clustered in the targets, but we have not rigorously examined clustering. A direct interaction between Whi3 and the UGCAU motif has not been demonstrated.

Second, FIRE found the additional motif AUUUUA, with a lower but still extremely high Z-score of 19.2. 29% of Whi3 targets have an AUUUUA in the 3′ UTR, while only 17% of non-targets do.

In mammals, a class of repeats called “AU Rich Elements” (AREs) is associated with mRNA instability [16], [17]. Although many distinct sequences can act as an ARE, the consensus sequence is AUUUA, which is quite similar to the AUUUUA motif found in the Whi3 targets. Interestingly, Vasudevan and Peltz studied ARE-mediated mRNA decay in *S. cerevisiae*, and found that the 3′ region of the *TIF51a* gene (aka *HYP2*), which encodes the translation factor eIF5A, contained a modular, transportable region specifying regulated mRNA turnover [18]. This module contained two repeats of AUUUA, but of great interest to us, it also contained two repeats of GCAU [18]. In fact, both *TIF51a/HYP2* and its homolog *ANB1* appear high on our list of Whi3 targets (ranks 8 and 12 on the joint list). Thus, one possibility is that Whi3 binds at GCAU motifs, but potentiates mRNA turnover (see below) through AREs such as AUUUA or AUUUUA. Many of the potential Whi3 targets, including *CLN3*, do contain strong AREs downstream of the GCAU motifs.

Because these motifs are short, we wondered whether Whi3 might recognize structure as well as sequence. Kertesz et al. defined RNA secondary structures genome-wide by using ribonucleases specifically recognizing single stranded or double stranded RNA structures [19]. They then created the searchable PARS database of yeast RNA structures. We searched this structure profile to see if any structural feature was associated with the UGCAU sites in Whi3 targets (Materials and methods), but did not find any such structure. For instance, the UGCAU is typically in single stranded RNA, but only to the same extent that other sequences are also typically single stranded. The UGCAU is not adjacent to a region that is preferentially double stranded or single stranded.

### Effects of Whi3 on RNA Expression and Half Life

Having found putative targets of Whi3, we next asked what effect Whi3 had on their expression. In principle, an RNA binding protein such as Whi3 could affect RNA stability; RNA storage; splicing (for those genes with introns); translation; and RNA localization. We first asked if deletion or over-expression of *WHI3* altered the steady-state levels of its target transcripts. Since over-expressing *WHI3* from the *GAL* promoter is lethal [1], we integrated three extra (four total) copies of *WHI3* at its genomic locus to create a strain constitutively over-expressing *WHI3*. This strain displayed a large cell size and was confirmed to have around a 4 fold increase in *WHI3* mRNA levels by Q-PCR. We called this strain “*WHI3*x4”. We then used microarrays to compare the levels of mRNAs in the *WHI3* wild-type, the *whi3* deletion, and the *WHI3*x4 over-expressor strain, and we did this in glucose medium and in YEP ethanol medium (Supplementary Table S3). Previously, Malcher et al. [20] also looked at *whi3* mutants (but not over-expressors) using microarrays. Their data has a correlation coefficient of about 0.42 with ours, and we have incorporated some of their data into our analysis (as noted below).

We note that the *whi3* mutant promotes Start, so *whi3* cells have relatively short G1 phases, and over-expression of *WHI3* delays Start, so *WHI3* over-expressors have long G1 phases. Therefore, genes that are differentially expressed between the *whi3* mutant and the *WHI3* over-expressor should include cell cycle regulated genes, but for an indirect reason.

We screened for genes that increased at least two-fold in the *whi3* glucose experiment, and that also increased in either the *whi3* ethanol experiment or the *whi3* experiment of Malcher et al., and also decreased in at least one of the *WHI3*x4 over-expressor experiments (glucose and ethanol). There were 34 genes that exactly or nearly met these requirements (Table 2). Of these, 8 (24%) were Whi3 RIP-chip targets, about a six-fold enrichment over the random expectation. These 8 included *YGR146c*, *MBF1*, and *SML1*, ranked 2, 5, and 10, respectively, on our list of 262 Whi3 targets. Also among the 34 genes was *PDR3*, which is not a highly-ranked target, but does contain an unusually high density of GCAU motifs. *PDR3* is a transcription factor, and the list of 34 genes also contains 10 genes that are normally induced by *PDR3*; possibly their abundance is increased in the *whi3* mutant (and decreased in the over-expressor) because the abundance of their transcription factor is increased. Finally, there are other genes of uncertain significance.

**Table 2:** Differentially regulated genes in *whi3.*

Common Name	SysName	*whi3* YPD	*whi3* YPE	*whi3* Mal.	*WHI3X4* YPD	*WHI3X4* YPE	Target Rank
*Up-regulated*							
PHO84	YML123C	3.7	0.0	0.2	−0.9	−0.4	17
RSB1	YOR049C	2.2		1.4	−1.1	−0.3	
PHM8	YER037W	2.5	−0.1	0.9	−1.5	−1.5	
YLR346C	YLR346C	2.7	1.2	0.0	−0.5	0.7	
DSE4	YNR067C	1.4	0.9	1.3	0.0	−0.5	212
GTO3	YMR251W	2.0	1.1	0.6	0.0	0.3	
FIT2	YOR382W	2.2	0.2	0.3	0.5	−0.6	
SPL2	YHR136C	1.9	0.4	0.3	−0.7	−1.1	203
YGR146C	YGR146C	1.8	1.1	0.3	0.2	−0.6	2
BDH2	YAL061W	2.3	1.1	−0.3	−0.5	0.0	
CHA1	YCL064C	1.1	0.9	0.9	−1.3	−2.4	
ATG7	YHR171W	1.2	0.4	0.6	0.1	−0.1	
CLB1	YGR108W	1.2	0.9	0.7	−1.2	−0.7	240
HES1	YOR237W	1.5	0.2	0.3	−0.2	−0.1	
GLK1	YCL040W	1.6	0.1	0.2	−0.1	−0.2	
MPM1	YJL066C	1.0	0.7	0.7	−0.3	−0.1	
SML1	YML058W	1.0	1.1	0.7	−0.7	−0.4	10
GDH3	YAL062W	1.4	0.6	0.3	−0.4	0.2	
MBF1	YOR298C-A	1.1	1.0	0.5	−0.5	−0.5	5
YHR140W	YHR140W	1.2	−0.2	0.5	−0.6	−0.1	
AIM17	YHL021C	1.3	0.8	0.3	−0.2	0.4	
ICY1	YMR195W	1.1	0.1	0.5	−0.4	−1.0	
GPD1	YDL022W	1.4	0.9	0.2	−1.7	−0.6	
ENO1	YGR254W	1.3	1.0	0.2	−0.4	−0.5	
YOR1	YGR281W	1.2	0.4	0.3	−0.3	−0.1	
HSP82	YPL240C	1.1	0.3	0.4	−0.6	−0.2	
GLO4	YOR040W	1.2	0.4	0.2	0.2	−0.6	
PDR3	YBL005W	1.2	−0.1	0.2	−0.1	−0.1	
SNQ2	YDR011W	1.1	0.6	0.3	−0.2	−0.2	171
PDR15	YDR406W	1.4	0.0	0.0	−0.7	0.0	
ICT1	YLR099C	1.1	0.0	0.0	−0.2	0.0	
VTC3	YPL019C	1.2	−0.1	−0.2	−0.2	−0.5	
CMK2	YOL016C	1.1	0.1	0.0	−0.2	−0.1	
YIL100W	YIL100W	1.0	0.7		−0.8	0.7	
*Down-regulated*							
SAM3	YPL274W	−1.4	−0.5	0.1	0.8	0.4	
GDH1	YOR375C	−1.4	−0.2	−0.1	0.3	0.9	
MMP1	YLL061W	−1.4	−0.4	−0.1	0.6	−0.1	
NDJ1	YOL104C	−1.9	NA	−0.4	0.6	0.2	
JHD2	YJR119C	−1.5	−0.2	−0.9	0.5	0.0	
POT1	YIL160C	−1.5	−0.6	−1.1	0.8	0.6	

We also looked for genes with the opposite behavior (i.e., low in the *whi3* mutant, high in the over-expressor), finding just 6 (Table 2). None of these were scored as Whi3 targets. Again, these genes are of uncertain significance.

These results suggest that the direct effect of Whi3 on mRNA steady state levels is relatively small in most cases, and also that many of the differentially-expressed genes seen by microarray analysis are differentially expressed because of secondary effects; i.e., they may not be direct targets of Whi3, since, typically, they do not co-precipitate with Whi3. However, the effects are not always small, since eight of the RIP-chip targets, including three of the ten highest-ranked targets, were also seen as differentially expressed genes. All of the RIP-chip Whi3 targets that were differentially expressed were increased in abundance in the *whi3* mutant and decreased in the Whi3 over-expressor, suggesting that Whi3 is reducing their abundance.

**Figure 1:**
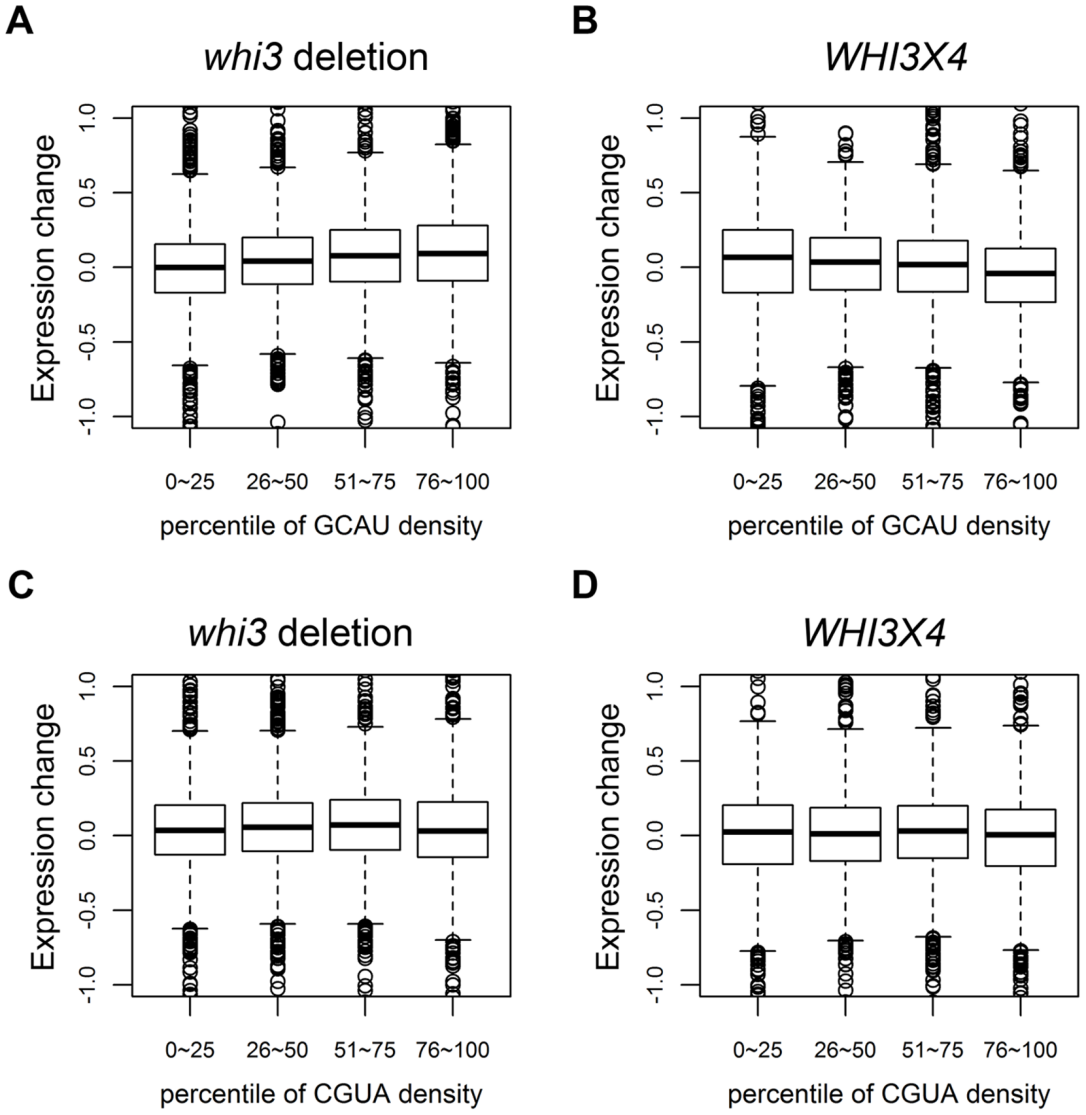
mRNAs rich in GCAUs have increased abundance in *whi3* mutants by microarray. Genes were divided into quartiles according to their GCAU density (Materials and methods). For each quartile, change in mRNA abundance is shown in (A) *whi3* mutants relative to wild-type; (B), *WHI3* over-expressors relative to wild-type. As a control, genes were divided into quartiles according to their CGUA density (an irrelevant control motif). For each quartile, change in mRNA abundance is shown in (C) *whi3* mutants relative to wild-type; and (D) *WHI3* over-expressors relative to wild-type. Changes are shown as the log2 of the ratio of abundance in *whi3* divided by abundance in WT (A, C) or the ratio of abundance in *WHI3X4* divided by abundance in WT (B, D).

To better analyze any small effect Whi3 might have on RNA levels genome wide, we divided all the mRNAs into four quartiles based on their GCAU density (Materials and Methods) and plotted the fold change in expression obtained from the microarrays for *whi3* and *WHI3*x4. A small but obvious trend was observed: in the *whi3* mutant, mRNA levels increased with increasing GCAU density (i.e., GCAUs per kilobase of the gene including UTRs), while in the *WHI3*x4 mutant, mRNA levels decreased (see Fig. 1), consistent with the idea that Whi3 might be destabilizing mRNAs that contain GCAU. The same analysis for 3′UTRs or coding regions separately gave the same result, and the same analysis for maximum local GCAU density (Materials and Methods) gave essentially the same result. Note that the effect was seen even when comparing the lowest quartile with the second lowest quartile–this suggests that Whi3 can act in proportion to GCAU density even for genes where that density is low. This also implies that Whi3 may be working on a very large number of mRNAs–perhaps thousands–to a very slight extent. No trend in RNA levels was observed for the density of the control tetranucleotide CGUA (Fig. 1). These results suggest that Whi3 has weak but wide-spread effects, and slightly reduces mRNA levels for its targets. This is consistent with the highly polymorphic phenotypes of *whi3*.

**Figure 2:**
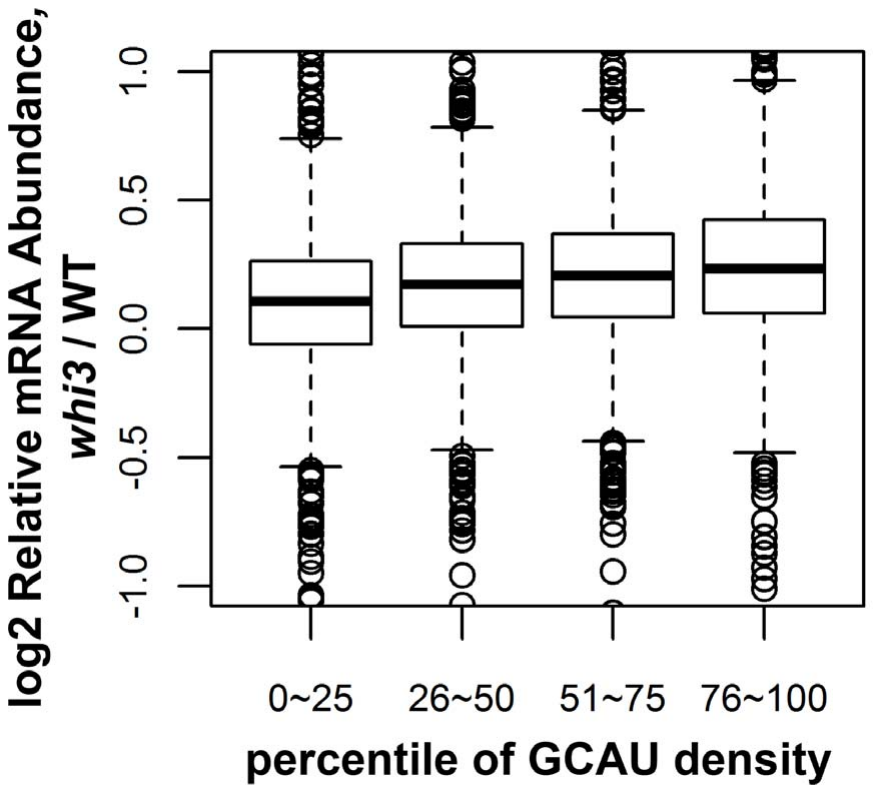
mRNAs rich in GCAUs have increased abundance in *whi3* mutants by RNA-Seq. Total RNA from *whi3* or WT cells was extracted and sequenced using a HiSeq. As in Fig. 1, genes were divided into quartiles according to their GCAU density (Materials and methods). For each quartile, relative mRNA abundance is shown for *whi3* mutants relative to wild-type as the log2 of the ratio of abundance.

Because these effects were small, we sought to confirm them using an assay other than the microarray. Therefore we used RNA-Seq to measure transcript abundance in a *whi3* mutant and a WT strain. Genes with higher GCAU density again showed slightly higher abundance in the *whi3* mutant strain than in the *WHI3* WT strain (Fig. 2) exactly consistent with the microarray results, and consistent with the idea that binding by Whi3 reduces mRNA abundance by some mechanism.

One explanation of the lower mRNA abundance of Whi3 targets would be that Whi3 decreases mRNA half-life. We attempted to examine this idea genome-wide using the temperature sensitive RNA polymerase mutant *rpb1-1* to turn off transcription, and then follow mRNA abundance with time using microarrays. In our first experiment, we did indeed see a statistically highly significant stabilization of many Whi3 target mRNAs in the *whi3* mutant (not shown). However, we could not reproduce this in two subsequent experiments (not shown). We have no explanation for the discrepancy between the experiments, except to say that the half-life measurements were quite noisy.

### Whi3 Reduces *CLN3* mRNA Levels

The main known functional target of Whi3 is the *CLN3* mRNA. Therefore we asked if Whi3 affects the abundance or stability of the *CLN3* mRNA.

**Figure 3:**
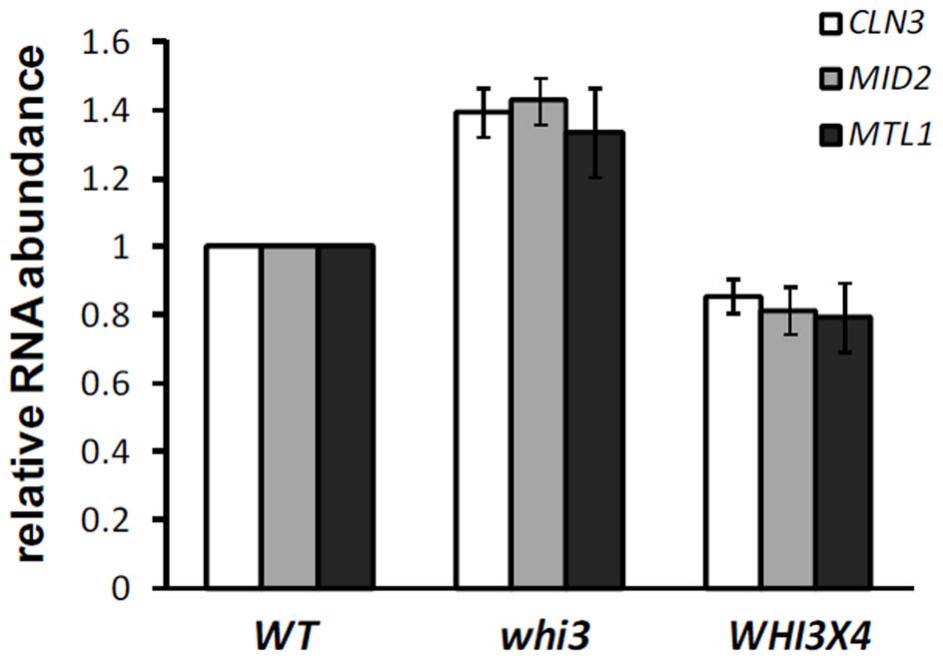
*CLN3* mRNA levels are increased in the *whi3* mutant. WT, *whi3* and *WHI3x4* cells were grown in YPD to log phase and total RNA was isolated and reverse transcribed into cDNA. RNA levels of *CLN3* and other two Whi3 targets, *MID2* and *MTL1,* were measure by Q-PCR and normalized using *ACT1*. The fold change in the abundance of *CLN3*, *MID2* and *MTL1* mRNAs in *whi3* and *WHI3x4* cells relative to WT cells is shown.

First, we tested Whi3’s effect on the abundance of *CLN3* mRNA. We used Q-RT-PCR to measure *CLN3* mRNA in total RNA isolated from a WT, *whi3* or *WHI3*x4 strain. The *ACT1* mRNA level was used for normalization. *CLN3* mRNA levels showed a small but significant increase of 1.4 fold in *whi3* mutants and a decrease of 0.8 fold in *WHI3*x4, compared to the level in WT cells. The same trend was observed for two other Whi3 target mRNAs, *MTL1* and *MID2*, in these PCR assays. (Fig. 3) Previous studies [2] reported no change in *CLN3* RNA levels in *whi3* mutant or Whi3 overexpression cells as assayed by northern blotting. The discrepancy can be explained by the small change and the higher precision provided by the Q-RT-PCR approach.

Although these changes are small, cell size is extremely sensitive to the dose of *CLN3* [3], and a 1.4-fold increase in *CLN3* abundance is phenotypically significant [3] (see Discussion).

Because the Whi3-dependent differences in *CLN3* mRNA were small, we also used a second approach, in which we compared the relative abundance of two different *CLN3* transcripts in the same cell, where one transcript contained the GCAU motifs for binding Whi3, and the other was a mutant transcript that did not. Colomina et al. have previously shown that the gcau mutant form of *CLN3* mRNA does not bind to Whi3 [4]. We constructed diploid strains that carried two, distinguishable forms of *CLN3* mRNA, a long form and a short form (Materials and Methods). In addition, we mutated all 12 GCAU motifs (the putative binding sites of Whi3) in the long or the short forms of *CLN3*, while preserving coding capacity (Materials and Methods). Thus, we had four versions of *CLN3*: the fully wild-type form (“Long-GCAU”); the long form lacking GCAU motifs (“Long-gcau_mut_”), the short form with GCAU motifs (“Short-GCAU”), and the short form lacking GCAU motifs (“Short-gcau_mut_”). These four forms of *CLN3* were inserted at the *CLN3* locus, expressed from the *CLN3* promoter, and characterized for function (Materials and Methods).

These haploids were then crossed to each other in various combinations so that alleles containing and lacking GCAU sites could be compared to each other when expressed in the same diploid cell. Using these diploids, in a single strain and a single RNA preparation, we could directly compare the abundance of the long form and the short form of *CLN3* by RT-PCR, and see whether the relative abundance depended on the presence of the GCAU motifs, and/or the *WHI3* gene.

Because the differences in abundance were small, we did 15 independent long:short ratio measurements in a *WHI3* strain, and 12 independent long:short ratio measurements in an isogenic *whi3* strain. Indeed, the ratios obtained depended on the presence or absence of the GCAU motifs; and also depended on the presence or absence of *WHI3*. The dependence on the presence or absence of Whi3 had a p-value less than 0.0001.

In a *whi3* mutant, where there is no Whi3 to interact with the GCAU motifs, loss of the GCAU motifs nevertheless caused a statistically significant decrease in the amount of *CLN3* mRNA (relative abundance of Long-gcau_mut_ = 0.46), presumably because one or more of the mutations has some Whi3-independent effect that decreases mRNA abundance. This could be due to some Whi3-independent sequence effect on mRNA stability, or it could be related to the fact that the 5′ GCAU motifs of *CLN3* overlap with the DDE motifs thought to affect *CLN3* expression [21]. In any case, this is a Whi3-independent effect, and the design of the experiment allows normalization for this effect.

In contrast, in a *WHI3* strain, loss of the GCAUs caused a statistically significant increase in the amount of *CLN3* mRNA (relative abundance of Long-gcau_mut_ = 1.15), consistent with the idea that loss of Whi3 binding stabilizes *CLN3*. That is, when normalized against WT mRNAs containing GCAUs, the mutant mRNAs lacking GCAUs increased 2.5-fold in abundance in *WHI3* strains vs *whi3* strains (1.15 divided by 0.46), consistent with the idea that Whi3 destabilizes the mRNA by about two-fold via the GCAU motifs (normalization method 1).

The key comparison was the relative amount of Long-GCAU *CLN3* mRNA in the *whi3* mutant versus the *WHI3* wild-type strain. This relative amount could be calculated by normalizing in each case to the amount of Short-gcau_mut_ *CLN3* (which should not be affected by the presence or absence of Whi3). This ratio was 1.50 (normalization method 2). That is, by this assay, fully wild-type *CLN3* mRNA is 50% more abundant in a *whi3* strain than in a wild-type strain. This is strikingly similar to the 1.4-fold increase seen in the straightforward quantitative PCR experiment (see above).

### Whi3 Destabilizes *CLN3* mRNA

**Figure 4:**
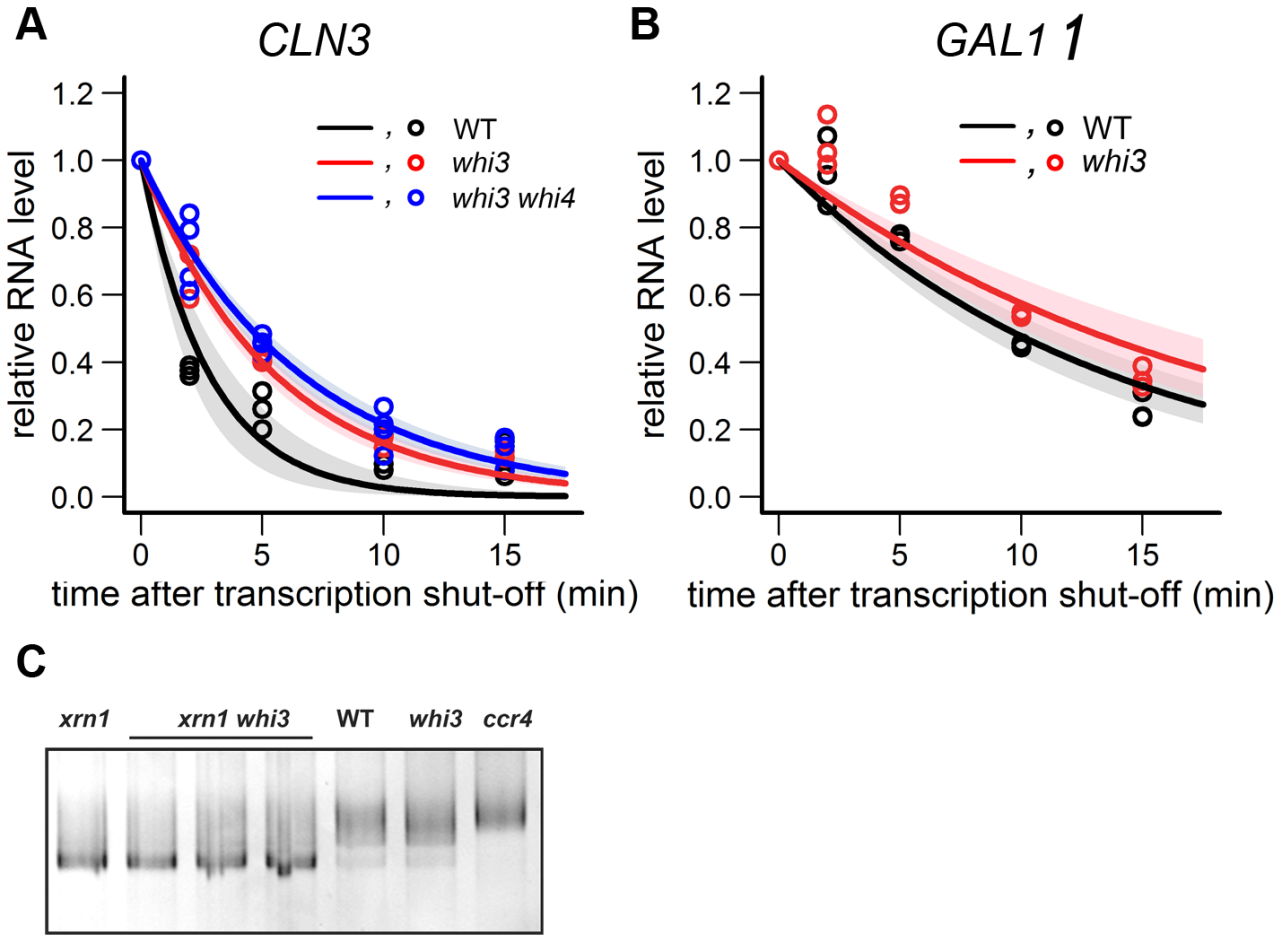
*CLN3* mRNA half-life is increased in the *whi3* mutant. *pGAL-CLN3 WHI3* (CYL104a), *pGAL-CLN3 whi3* (CYL105a), and *pGAL-CLN3 whi3 whi4* (CYL133a) cells were grown in YP medium with 2% raffinose and 2% galactose to log phase. 30 ml of culture was collected as t_0_. Glucose was added to a final concentration of 2% to stop *CLN3* transcription. Cells were collected at 2, 5, 10 and 15 min. (A) *CLN3* mRNA levels and (B) *GAL1* mRNA levels were measured by Q-PCR and normalized using *ACT1* for each sample. Lines show a first order rate fit of the decay data. Shaded regions indicate the 95% confident interval of the fit. (black, WT; red, *whi3*; blue, *whi3 whi4*) Decay of *GAL1* is a control. (C)LM-PAT analysis of *CLN3* poly(A) tail length in *xrn1* (lane 1), *xrn1 whi3* (lane 2–4), WT (lane 5), *whi3* (Lane 6) and *ccr4* (lane 7).

To directly measure *CLN3* mRNA half life, we inserted a *GAL* promoter in front of *CLN3*. Cells were grown in medium with raffinose and galactose to mid log phase. The *GAL* promoter was then repressed by addition of glucose, and *CLN3* mRNA levels were followed by Q-PCR. In WT cells, *CLN3* mRNA decreased by 60% at 2 min, while in *whi3* cells the decrease was only 40% (Fig. 4). We fit the data to a first order rate kinetic model of decay. The estimated half life for *CLN3* was 1.9 min in WT cells (consistent with the 2 min half-life previously reported [22], [23], [24]) and 3.8 min in *whi3* cells, suggesting a two-fold stabilization of *CLN3* RNA in *whi3* mutant cells. In contrast, the half-life of a control mRNA, *GAL1*, did not change in the *whi3* mutant.

Whi3 has a homologue named Whi4. Although the *whi4* mutant shows only a very small decrease in cell size, the *whi3 whi4* double mutant is noticeably smaller than either single mutant, suggesting redundancy [1]. Therefore, we also measured *CLN3* mRNA half-life in the *whi3 whi4* double mutant. As shown in Figure 4, there was a slight increase in *CLN3* mRNA half life, to about 4.5 min. Although the various mRNA half-life differences are small, they were repeatable over multiple experiments (Fig. 4), and the 95% confidence interval for the half-life in the WT strain is clearly separated from the 95% confidence interval in the *whi3* or *whi3 whi4* strains (Fig. 4).

In the above *GAL1*-*CLN3* experiments, the 5′UTR of *CLN3* was replaced by the 5′UTR of *GAL1*. However, the *CLN3* 5′UTR contains *cis* translational regulatory elements which may also affect RNA stability as a side effect of translation [25], [26], [27]. Furthermore, of the 12 GCAU sites in *CLN3* mRNA, six are in the 5′UTR while the remaining six are towards the 3′ end of the coding region. Therefore, we made a construct in which the *GAL* promoter was inserted further upstream of the *CLN3* ORF, preserving the endogenous *CLN3* 5′UTR. The *CLN3* RNA half life was measured as described above. Again, the *CLN3* RNA was more stable in the *whi3* mutant than in WT cells, this time by 1.5 fold (4.4 min in *whi3* cells vs 2.9 min in WT). Thus, the GCAU motifs in the 5′ UTR may not be important for the destabilization of *CLN3* mRNA, even though it is known that they can mediate binding [4] (see Discussion).

Whi3 physically interacts with Not1, Not3, Not4, Caf40, and Pop2 [28], which are all subunits of the Ccr4-Not complex, the predominant deadenylase in the cytoplasm [29], [30], [31]. Shortening of poly(A) tails of the mRNA is an initial step of RNA decay [32], [33], [34], [35], [36]. To see if Whi3 affects the half lives of its target RNAs at the step of poly(A) tail length, we measured the poly(A) tail length of *CLN3* RNA in WT and *whi3* cells using the ligation-mediated poly(A) test (LM-PAT) [37], [38]. In this PCR based method, a shift in the mobility of the PCR product indicates a change in the length of the poly(A) tail. As shown in Figure 4c (lane 5–7), no difference in the poly(A) tail length of *CLN3* was observed in the *whi3* mutant, though accumulation of longer poly(A) tracts were detected in the positive control, the *ccr4* mutant, as expected.

It was possible that the inability to detect *CLN3* mRNAs with very short tails was due to their extremely rapid degradation. Therefore, we repeated the experiment in an *xrn1* background. *XRN1* encodes the exonuclease degrading RNA from 5′ to 3′ [33], [34], [39], [40]. Deletion of *XRN1* blocks 5′ to 3′ degradation and allows accumulation of RNAs with short poly(A) tails. In the *xrn1* background, all the RNAs tested showed a higher mobility band, indicating accumulation of short poly(A) tail RNA. However, the level of accumulation was similar in WT and *whi3* mutant (see Figure 4c, lane 1–4). We therefore see no evidence that Whi3 destabilizes *CLN3* mRNA by regulating poly(A) tail length. However, the assay was a qualitative one (i.e., looking at a gel).

We also tested *CLN3* mRNA half life using the same galactose inducible system as described above in the *xrn1* deletion background. In the *xrn1* mutant, *CLN3* mRNA was dramatically stabilized and its half life increased by around 12 fold to 23.5 min. This suggests that after deadenylation, *CLN3* is degraded in a 5′ to 3′direction through decapping. No difference was observed for the *CLN3* mRNA degradation curve between the *xrn1* single mutant and the *whi3 xrn1* double mutant and the half life found in the double mutant was 23.2 min. Although several interpretations are possible, the most obvious is that *CLN3* mRNA degradation requires the Xrn1 pathway, whether that degradation is mediated by Whi3 or not.

### Ribosome Profiling

Ribosome profiling is a relatively new technique that footprints actively translating ribosomes, providing a record of their positions on all mRNAs [41], [42], [43]. The total number of footprints for a particular species of mRNA gives the number of ribosomes engaged in translating that mRNA.

Because the binding of Whi3 to an mRNA could alter translation, we used ribosome profiling on *whi3* and wild-type strains. More than 14 million footprint reads were obtained for each strain. Full results are available at NCBI’s GEO database under accession number GSE51164.

**Figure 5:**
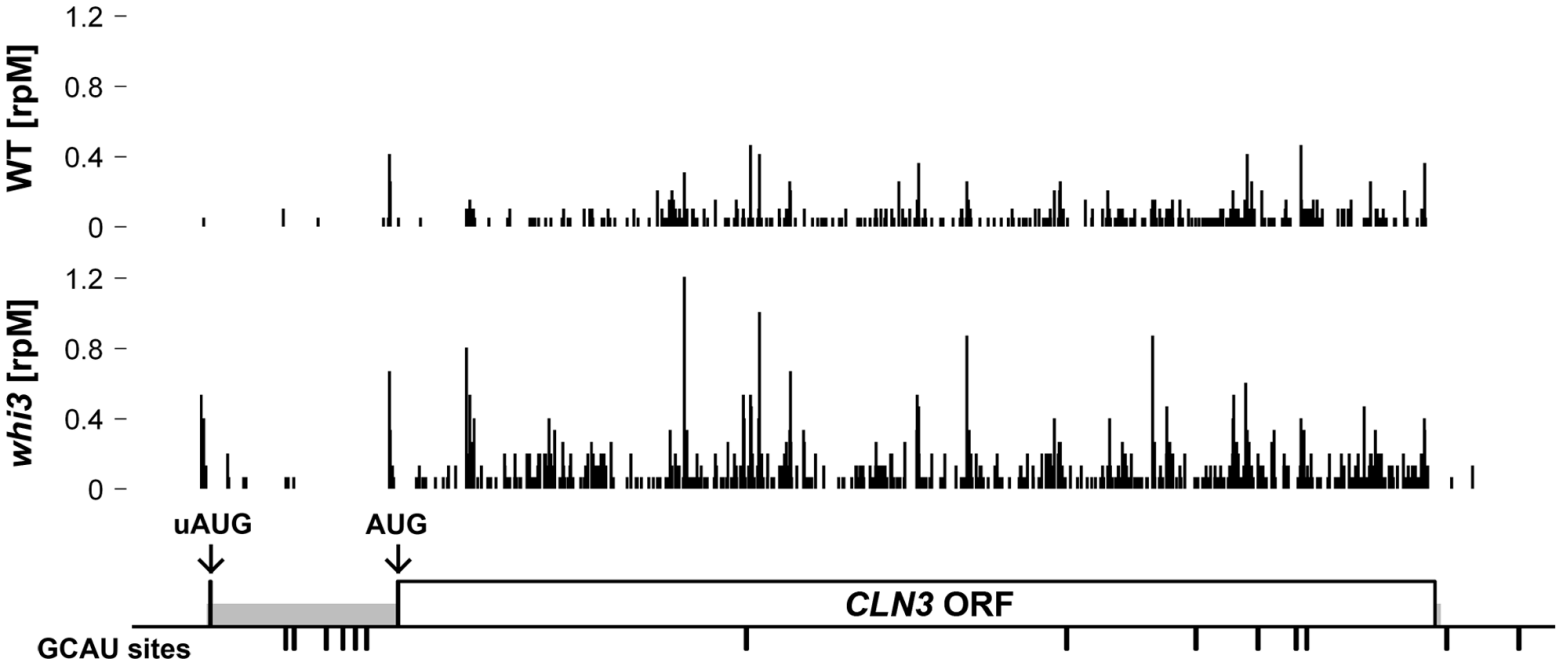
Ribosome profile of *CLN3* mRNA. Ribosome densities in the *CLN3* region are shown for WT (upper) and *whi3* (middle) cells. The x-axis shows the footprint position relative to the sense strand of *CLN3*. The y-axis shows the read count for *CLN3* normalized by total read count for all mRNAs (rpM is “reads per million reads”). The greater number of ribosome footprints over *CLN3* in the *whi3* strain (2.3 fold) is partly due to a greater abundance of *CLN3* mRNA (1.5 fold) (as determined in the RNA-Seq part of the experiment), and partly due to an increased translational efficiency (1.5 fold). The bottom panel shows genomic features of *CLN3*. The 5′UTR and 3′UTR are shaded in gray. The arrows show the position of the upstream AUG (uAUG) and the start AUG of the ORF. The ticks show the position of GCAU sites.

The most striking observation was that in the *whi3* mutant, there were 2.3 fold more ribosome footprints over *CLN3* than in the wild-type (Fig. 5). That is, there were 2.3 fold more ribosomes passing over *CLN3* mRNA in *whi3* cells, suggesting that about 2.3 fold more Cln3 protein would be produced. This number was obtained by normalizing to all other ribosome footprints for all other genes, by the standard “reads per kilobase per million mapped reads” method (RPKM).

There were two contributors to this 2.3 fold increase. First, the RNA-Seq portion of the experiment showed that the *CLN3* mRNA was 1.52 fold more abundant in the *whi3* strain than in the wild-type strain. This increased abundance is nearly identical to that measured from Q-PCR and from the long-short GCAU comparison. This measurement is quite accurate, because there were hundreds of reads of *CLN3* in each arm of the comparison.

**Figure 6:**
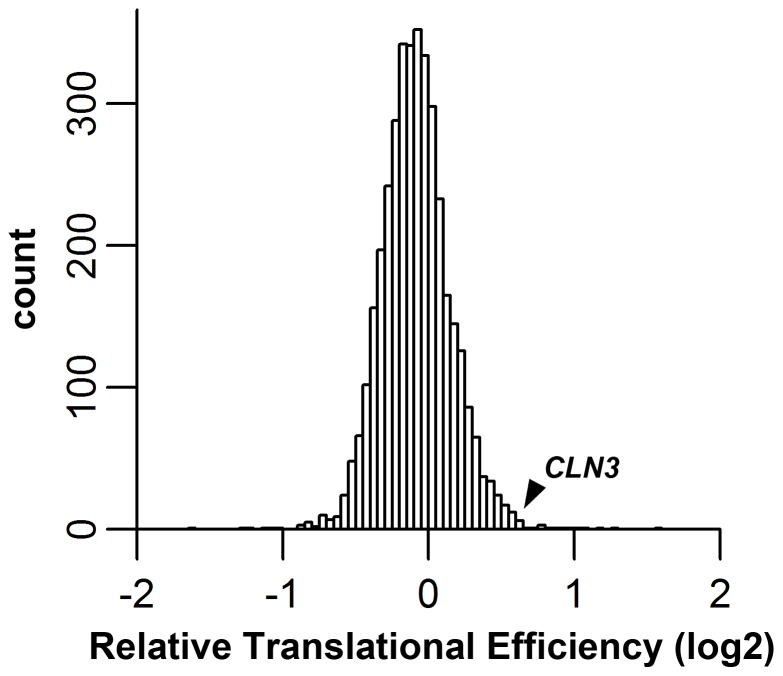
Distribution of relative translational efficiencies in *whi3* vs WT. Translational efficiency (the number of ribosome footprints per mRNA, showing ribosome occupancy, divided by the number of RNA-Seq reads, showing mRNA abundance) was calculated for each gene. For each gene, the ratio of translational efficiency in *whi3* cells to translational efficiency in WT cells was calculated. The log2 of this ratio is displayed. A value of 0 indicates that translational efficiency is the same in *whi3* and WT cells. The position of *CLN3* is indicated by the arrow; *CLN3* has a relative translational efficiency of 1.5. This is statistically different from 1 (p<0.002).

Second, *CLN3* had a higher translational efficiency (i.e., more ribosome footprints per *CLN3* mRNA) in the *whi3* strain than in the wild-type strain (Fig. 5). The translational efficiency was 1.53 (i.e., about 50% more ribosome footprints per *CLN3* mRNA in *whi3* than in WT). This measurement is also quite accurate, again because of a large number of footprint reads. We calculated the relative translational efficiencies for all genes in *whi3* vs wild-type (Fig. 6), and found that the *CLN3* gene had the 19^th^ highest relative efficiency in the genome. This was highly significant (p-value of 0.002 for a one-tailed test, ignoring genes with less than 200 sequence reads). Like *CLN3*, many of the other genes with a high relative translational efficiency in *whi3* mutants had GCAU motifs close to the Start codon (in some, the “UG” residues of the AUG Start codon were part of a UGCAU motif); however, we have not yet calculated the statistical significance of this effect.

Thus, there were about 1.5 fold more *CLN3* mRNAs in the *whi3* mutant, and each of these mRNAs was translated by about 1.5 fold more ribosomes, leading to a total increase of about 2.3 fold.

Other observations from the ribosome profiling include the fact that there is no obvious correlation between the pattern of ribosome footprints (the peaks and valleys) and the position of GCAU motifs (Fig. 5). We note that peaks and valleys generally like those in *CLN3* are also seen in most other genes. Also, in the *whi3* mutant, there is a pronounced peak of footprints at the upstream AUG at about -315 in the 5′ UTR, whereas this peak is almost completely absent in the WT strain. The upstream AUG has been implicated in translational control of *CLN3* under certain nutritional conditions [26]. Although our results come from only one measurement, it is possible that Whi3 and the six GCAU motifs in the *CLN3* 5′ UTR are somehow inhibiting translation initiation at the *CLN3* start codon, and this explains the change in translational efficiency and is somehow reflected in the preference for the upstream AUG versus the downstream start AUG. Additional experiments will be required to investigate this hypothesis.

### Whi3 Co-localizes with P-bodies/Stress Granules

Gari et al. [2] used immunofluorescence to show that Whi3 has a somewhat lumpy cytoplasmic localization. This led to the hypothesis that cytoplasmic aggregates or foci of Whi3 were restraining Cdc28-Cln3 complexes from entering the nucleus, thereby inhibiting it [5]. Wang et al. [5] showed that Whi3 physically interacts with Ded1, an RNA helicase essential for translation initiation. Ded1 is a component of P-bodies [44], which are cytoplasmic granules involved in processing and degradation of mRNAs. Tarassov et al. [28] also found evidence of a physical interaction between Whi3 and several P-body components, including Ded1, Pub1, Scd6, Dhh1, and Lsm4. We were interested in seeing the localization of Whi3 in live cells, and whether it had any relationship to P-bodies.

**Figure 7:**
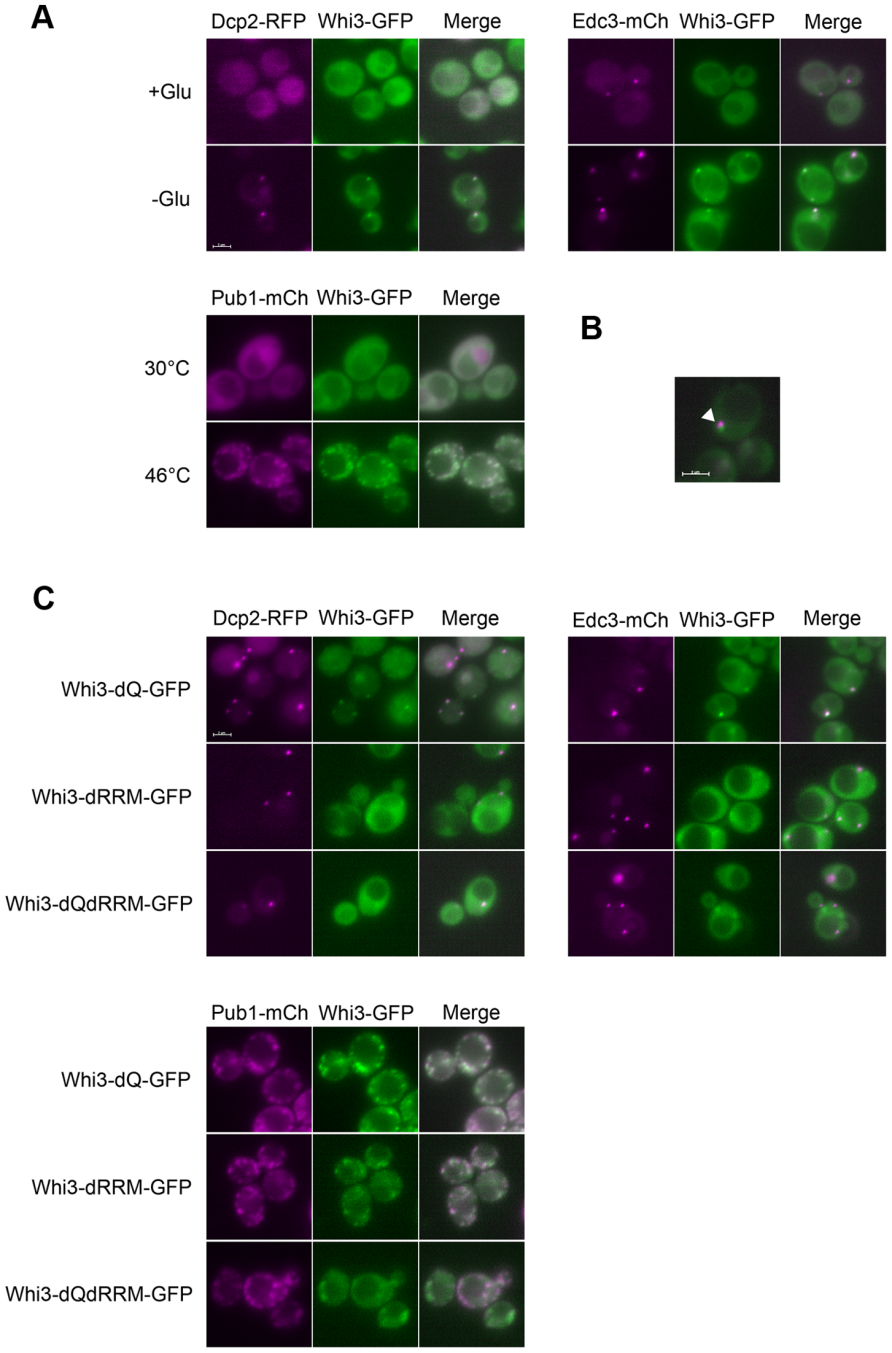
Whi3 co-localizes with P-bodies/stress granules. (A) Upper: Co-localization of chromosomal GFP tagged Whi3 (green) with P-body markers: Dcp2-RFP (pRP1155, magenta) and Edc3-mCherry (pRP1574, magenta) during log phase growth (+Glu) or after 30 min of glucose starvation (-Glu). Lower: Co-localization of chromosomal GFP tagged Whi3 (green) with stress granule marker Pub1-mCherry (pRP1661, magenta) after 10 min shift to 46°C. (B) Co-localization of Edc3-mCherry (magenta) and Whi3-GFP (green) after 30 min of glucose starvation. A partial co-localization is shown by the arrow. (C) Co-localization (or lack thereof) of GFP-tagged *whi3* rrm or Q-domain mutants with P-body markers under stress. Upper: 30 min of glucose starvations. P-body markers are Dcp2-RFP (pRP1155, magenta) and Edc3-mCherry (pRP1574, magenta). Lower. 10 min of heat shock at 46°C. Stress granule marker is Pub1-mCherry (pRP1661, magenta). A scale bar of 2 µm is shown in each panel.

A Whi3-GFP fusion was created, which has fully wild-type function as assayed by cell size. When cells are grown in glucose, a condition in which the small cell phenotype of *whi3* is manifested, Whi3-GFP is somewhat unevenly spread throughout the cytoplasm (Fig. 7). Although its distribution is not completely homogeneous, it does not appear lumpy, or to be in distinct foci. Its unevenness could be due to exclusion from intracellular compartments such as the nucleus and vacuole.

We asked if Whi3 co-localizes with P-bodies. To identify P-bodies, we used Dcp2-RFP (from Dr. R. Parker, Materials and Methods). Dcp2 is an RNA decapping enzyme shown to be a component of P-bodies [45], [46]. Cells growing in medium containing 2% glucose showed neither Whi3-GFP foci nor Dcp2-RFP foci, consistent with the observation that few or no P-bodies are seen in glucose-fed, exponentially growing cells [47]. However, after glucose was depleted for 30 min, both Whi3-GFP and Dcp2-RFP foci became visible and Whi3-foci co-localized with Dcp2-RFP foci. (Figure 7a) We obtained the same result for another P-body marker, Edc3-mCherry, which also plays a role in RNA decapping [45], [48]. Unlike Dcp2, Edc3-mCherry foci were sometimes visible even in the presence of glucose, but the number was typically 0 to 2 per cell. In response to glucose depletion, the number of Edc3-mCherry foci increased dramatically, and Whi3-GFP foci then became visible and co-localized with Edc3-mCherry foci. The co-localization was not perfect: there were occasional foci containing either Whi3 or the P-body marker, but not both, and in addition there were examples of incomplete overlap between the markers (e.g. Fig. 7b).

Stress granules are cytoplasmic foci related to P-bodies involved in storage and translation repression of RNA and induced under various stress conditions. Stress granules share some protein components with P-bodies [49] [50]. We also asked if Whi3 co-localizes with stress granules using Pub1-mCherry as the stress granule marker under heat shock conditions (however, Pub1 may not be completely specific for stress granules) [51], [52] [50]. As shown in figure 7a, both Whi3-GFP and Pub1-mCherry were distributed homogenously throughout the cytoplasm during exponential, unstressed growth. 10 minutes after a 46°C heat shock, both Whi3-GFP and Pub1-mCherry formed foci, and they usually (but not always) co-localized. These foci formed under heat shock were larger and more numerous than those formed under glucose starvation, and some foci were apparently connected to each other. We have not attempted to rigorously distinguish P-bodies from stress granules, but it appears Whi3 is associated with both.

Whi3 is not required for the formation of P-bodies/stress granules, since Dcp2-RFP foci still form normally in a *whi3* mutant in response to glucose depletion (data not shown).

Since Whi3 was not required for P-body/stress granule formation, we asked what recruits Whi3 to these foci. Whi3 protein has two prominent domains: the Q-rich domain (rich in glutamine) and the RRM (RNA-binding) domain [1]. It has been proposed that Q/N rich domains are prion-like and have a role in protein aggregation. Many known P-body components contain Q/N rich domains, and it has been shown that these domains either enhance the accumulation of the protein in the P-body or promote the formation of the P-body or aggregation [53], [54]. To see if the Q-rich domain functions in the accumulation of Whi3 in the P-body we deleted amino acids 201∼300 (the Q-rich region) in Whi3-GFP, to yield Whi3-dQ-GFP, and tested the co-localization of Whi3-dQ-GFP with P-bodies and stress granules as described above. Deletion of the Q-rich domain did not affect co-localization of Whi3 with P-bodies or stress granules. (e.g., Figure 7c).

Next, we deleted the C-terminal region of Whi3 from aa 539 onwards to delete the RNA binding domain (RRM) and asked if the RRM domain plays a role in recruiting Whi3 to cytoplasmic foci. After glucose depletion, the co-localization of Whi3-dRRM-GFP with a P-body marker was largely but not entirely disrupted. However, after heat shock, co-localization was still observed, but the Whi3-dRRM-GFP was apparently less concentrated in foci (Figure 7c). Finally, we deleted both the RRM and the Q-rich domains. Now, few if any distinct Whi3 foci were observed even after glucose depletion or heat shock, despite wild-type protein abundance (data not shown). To the extent that occasional concentrations of Whi3-dQ-dRRM-GFP were seen, they were diffuse, and not well co-localized with P-bodies or stress granules. This result suggests that the Q-rich domain and the RRM domain are redundant in localizing Whi3 to P-bodies/stress granules.

**Figure 8:**
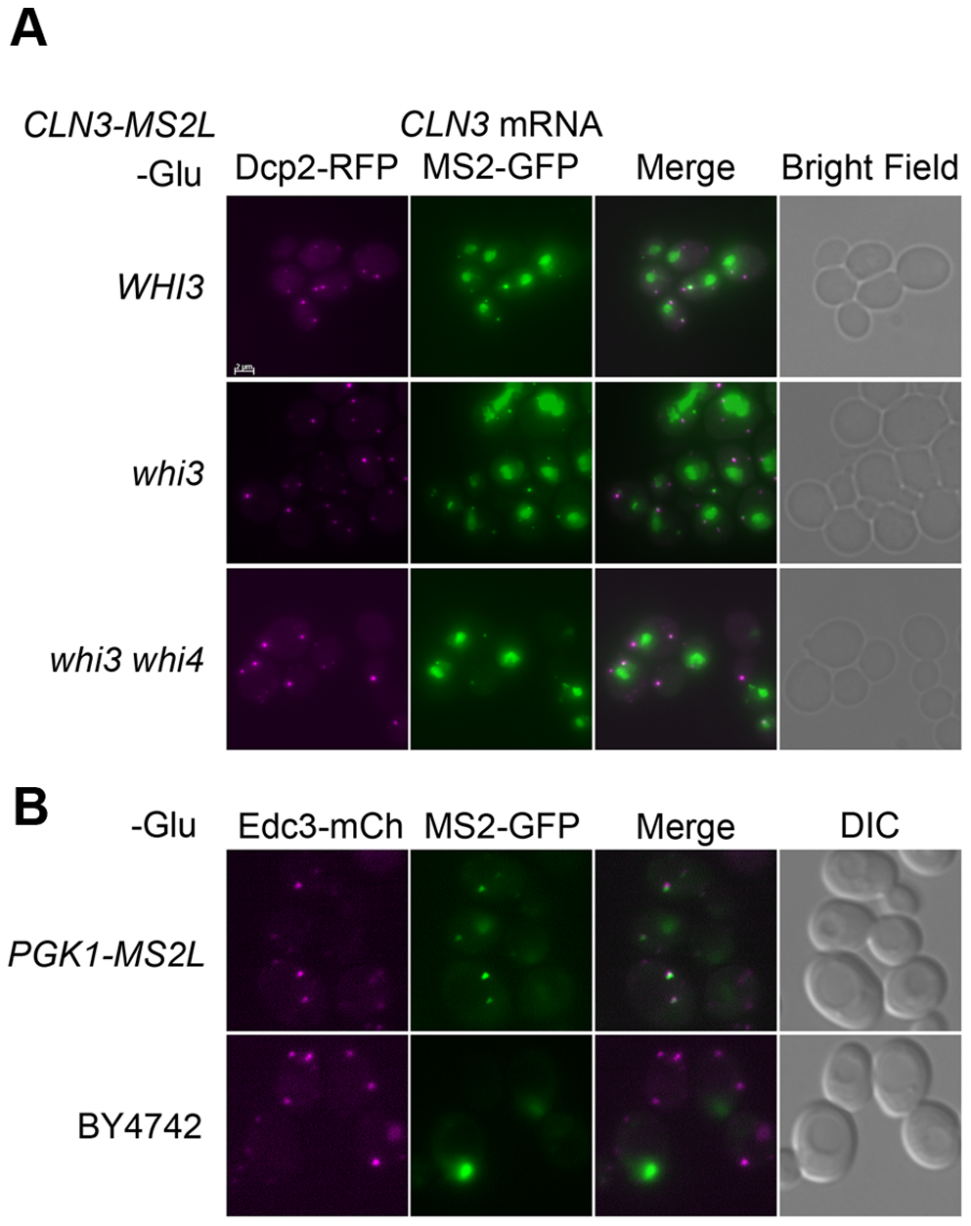
Under stress, *CLN3* mRNA co-localizes with P-bodies/stress granules. (A) Cells with MS2 loop tagged *CLN3* in *WHI3*, *whi3* or *whi3 whi4* were cotransformed with Dcp2-RFP (pRP1155-LYS2) and MS2-GFP (YCplacIII-MS2-GFP) for co-localization of P-bodies and *CLN3* mRNA. Cells were starved of glucose for 30 min. Dcp2 is shown in magenta and MS2-GFP is shown in green. Excess MS2-GFP in the nucleus appears as large green lumps. Small green foci indicate *CLN3* mRNA. Micrographs were taken as Z-series and the images are shown as a projection of z-stacks. At higher contrast and brightness, additional *CLN3* foci can be seen in some cells. (B) Upper. Cells with MS2 loop tagged *PGK1* (PGK1-MS2L) were cotransformed with Edc3-mCherry (pRP1574) and MS2-GFP (YCplacIII-MS2-GFP). Cells were starved of glucose for 30 min. Edc3-mCherry is shown in magenta and MS2-GFP is shown in green. Lower. Control cells with no MS2 loops (BY4742) were transformed with Edc3-mCherry (pRP1574) and MS2-GFP (YCplacIII-MS2-GFP). No cytoplasmic GFP foci are seen. A scale bar of 2 µm is shown in panel A.

Since Whi3 selectively binds a subset of RNAs and co-localizes with P-bodies/stress granules, Whi3 might bring its bound RNAs to P-bodies/stress granules. To test this hypothesis, we examined the *in vivo* localization of *CLN3* mRNA using the MS2 system [55]. We integrated 12 copies of MS2 loops between the coding region and the 3′UTR of *CLN3* RNA and ectopically expressed NLS-MS2-GFP, which binds specifically to the MS2 loops. The NLS in MS2-GFP sequesters the unbound MS2-GFP in the nucleus so that the GFP background is reduced in the cytoplasm. (reagents obtained from [55], [56]). Cells containing *CLN3* with 12 MS2 loops were wild-type for cell size, showing that the gene is functional. In cells carrying *CLN3* with the MS2 loops, but not in control cells with *CLN3* lacking the loops, one or two bright GFP foci (and sometimes additional less bright foci) were observed in the cytoplasm (Fig. 8). The much larger, brighter green spots are nuclei, which contain unbound NLS-MS2-GFP. We co-expressed Dcp2-RFP with NLS-MS2-GFP in the *CLN3*-MS2 tagged cells, and observed that, under glucose starvation, the GFP foci containing *CLN3* mRNA co-localized with Dcp2-RFP foci, suggesting that some of the *CLN3* mRNA was localized to P-bodies/stress granules (Fig. 8). Perhaps surprisingly, the co-localization of *CLN3* mRNA and the P-body marker was preserved in the *whi3* mutants and in the *whi3 whi4* double mutant, which implied that neither Whi3 nor Whi4 is required for localizing *CLN3* mRNA to the P-body. Additionally, we found that *TUB2* and *PGK1*, which do not bind Whi3 as far as we know, also co-localized with the Dcp2 foci in response to glucose depletion (data not shown and Figure 8b). Thus, although Whi3 and its target *CLN3* can both be found in P-bodies/stress granules under stress conditions, it is unclear what the role of Whi3 is, if any, in targeting *CLN3* to this location.

## Discussion

### The Whi- Phenotype of *whi3* Mutants is due to Stabilization, Increased Abundance, and Increased Translational Efficiency of *CLN3* mRNA

The *whi3* mutant was found because it has small cells. Nash et al. [1] found that a *whi3* mutant has a mode cell volume of 35 femtoliters, versus 45 femtoliters for a WT cell. Interestingly, Nash et al. [3] found that a strain with two copies of the *CLN3* gene also has a mode cell volume of 35 femtoliters, versus, in this case, 44 femtoliters for a WT cell. That is, a doubling of the *CLN3* dosage gives exactly the same phenotype as the *whi3* mutation. Here, six different experiments all showed that the *whi3* mutation increases the abundance or stability of the *CLN3* mRNA. The estimated increases were 1.4 fold (Q-PCR); 1.5 fold (long-short ratio measurement, normalization method 2), 1.5 fold (RNA-Seq); 1.5 fold (stabilization in *GAL-CLN3* experiment with 5′ UTR), 2 fold (stabilization in *GAL-CLN3* experiment without 5′ UTR) and 2.5 fold (long-short ratio measurement, normalization method 1). Over the six kinds of experiments, the median increase in *CLN3* abundance in the *whi3* mutant is 1.5 fold, and the mean increase is 1.7 fold, which is almost enough to explain the *whi3* small-cell phenotype [3].

But in addition, it appears that Whi3 inhibits the translation of *CLN3* (Fig. 5, 6), possibly because of the 5′ cluster of GCAU sites in the 5′ UTR (Fig. 5). The increased translational efficiency of *CLN3* in the *whi3* mutant is 1.5 fold. Together, the increased *CLN3* mRNA abundance/stability (1.5 to 1.7 fold), and the increased translational efficiency (1.5 fold) should give about 2.3 fold more protein and therefore are fully sufficient to explain the *whi3* small cell phenotype, which requires a two-fold increase in Cln3 amount [3]. Possibly the destabilization of *CLN3* depends mostly on the 3′ GCAU motifs, and the inhibited translation of *CLN3* depends mostly on the 5′ GCAU motifs; further experiments will be required to test this possibility.

### Whi3 Destabilizes Many mRNAs

Microarrays, RNA-Seq, and other methods show that the Whi3 protein reduces the mRNA abundance of its RIP-chip targets and of many other genes that contain GCAU motifs. At least in the case of *CLN3*, reduced abundance is because of reduced half-life. The effects are probably very widespread. Figs. 1 and 2 show that the reduction is proportional to the density of GCAUs. On its own, this could mean that many, most, or virtually all mRNAs could be affected to varying degrees, or it could mean that a small number of mRNAs are affected very strongly, and these few mRNAs change the average. However, the second interpretation is less consistent with the fact that by microarray, only modest effects are seen. Furthermore, evidence from RNA-Seq shows directly that hundreds of mRNAs are being affected, with almost all of the effects quite small.

mRNA turnover is thought to begin with gradual deadenylation of the poly(A) tail [29], [30], [31]. This deadenylation is carried out by the Ccr4-Not complex, a large complex with many functions, but which includes two 3′ exo-ribonucleases, Ccr4 and Caf1 (in *S. cerevisiae*, Caf1 is thought to be catalytically inactive) [29], [30], [31]. By poorly characterized mechanisms, but potentially through interactions with sequence-specific RNA binding proteins, the Ccr4-Not complex is thought to be brought preferentially to some mRNAs, and thereby preferentially shortens the poly(A) tail of these mRNAs. Once the poly(A) tail has been shortened to some critically short length, the 5′ cap on the mRNA is removed by a decapping enzyme, and this allows rapid degradation from the 5′ end via a 5′ exo-nuclease of the Xrn1 family. In this model, mRNA half-life would be largely determined by the extent to which the Ccr4-Not complex is attracted to a specific mRNA.

Tarassov et al. used a Protein Complementation Assay to find yeast protein-protein interactions on a genome-wide scale [28]. Strikingly, they found that Whi3 interacts with five components of the Ccr4-Not complex, and Whi4 also interacts with four of these. Whi3 also interacts with four other proteins affecting mRNA turnover. All of the physical interactions found for Whi3 and Whi4 are shown in Table 3. These protein-protein interactions of Whi3 and Whi4 are supportive of a model in which the binding of Whi3 to an mRNA attracts the Ccr4-Not complex, which then accelerates mRNA degradation. We failed to observe the poly(A) shortening expected from this model (Fig. 4), but the expected change is small and the assay was not quantitative.

**Table 3:** Proteins Interacting with Whi3.

Bait	Interactor	Function
Whi3	Whi3	RNA binding protein
	Pub1	RNA binding protein involved in mRNA turnover
	Puf4	RNA binding protein involved in mRNA turnover
	Not4/Mot2	Ccr4/Not complex. Involved in mRNA turnover. Ubiquitin ligase.
	Not1/Cdc39	Ccr4/Not complex. Involved in mRNA turnover.
	Not3	Ccr4/Not complex. Involved in mRNA turnover.
	Caf40	Ccr4/Not complex. Involved in mRNA turnover.
	Pop2	Ccr4/Not complex. Involved in mRNA turnover.
	Lsm4	RNA binding complex involved in RNA turnover, decapping.
	Dhh1	RNA helicase, stimulates decapping; interacts with Ccr4/Not
	Ded1	RNA helicase needed for initiation of translation.
	Scd6	Binds eIF4G; represses initiation of translation.
	Def1	Factor for degradation of RNA polymerase II
	Mbf1	Transcriptional co-activator; also found as mRNA target of Whi3
Whi4	Whi3	RNA binding protein.
	Not4/Mot2	Ccr4/Not complex. Involved in mRNA turnover. Ubiquitin ligase.
	Not1/Cdc39	Ccr4/Not complex. Involved in mRNA turnover.
	Not3	Ccr4/Not complex. Involved in mRNA turnover.
	Caf40	Ccr4/Not complex. Involved in mRNA turnover.

Interaction data is taken from Tarassov et al. [28], and shows all proteins found to be interacting with Whi3 or Whi4 baits.

Interestingly, Tarassov et al. also found an interaction between Whi3 and Ded1 and Scd6 [28]. Ded1 is needed for initiation of translation, and Scd6 is a direct inhibitor of initiation of translation. This interaction could help explain how Whi3 reduces translational efficiency of *CLN3* and perhaps some other mRNAs.

Our overall model for Whi3 is that it binds to GCAU motifs on mRNAs. In many cases, particularly if the GCAUs are towards the 3′ end of the mRNA, or if an AU Rich Element (ARE) is present, the bound Whi3 might attract the Ccr4-Not complex, which would increase the rate of deadenylation and mRNA turnover. On the other hand, if the GCAUs are near the start codon, there could be other effects. In *CLN3*, there are six GCAUs in the 5′ UTR, and it appears that Whi3 inhibits translation. This could be by physical occlusion of the ribosome from the start codon; alternatively, the interaction of Whi3 with Ded1 or Scd6 could lead to inhibition of translation. We note that the function of another RNA binding protein, Mmi1, varies according to where it binds in its target mRNA [58]. Binding near the 3′ end leads to rapid RNA degradation; binding further from the 3′ end results in slower RNA degradation; and binding near a splice site inhibits splicing. Similarly, it is possible that Whi3 functions in different ways depending on the context of binding.

We have also shown that Whi3 is a component of P-bodies, but most of our experiments are in conditions where P-bodies are not seen. Accordingly, we have little idea of the role of Whi3 in P-bodies. However, the presence of Whi3 in P-bodies could possibly be connected to the previous finding of cytoplasmic foci containing Whi3 [2], [5].

### Small Effects

Perhaps surprisingly, the effects we have observed of Whi3 on target RNAs are all small, in the range of two-fold or less. Although for some mRNAs, such as *CLN3*, a two-fold effect is large enough to have major phenotypic effects [3], in general one has to wonder about the biological reasons for effects of this size (see below). At the same time, small effects are difficult to demonstrate rigorously. In the case of *CLN3*, we have used several different kinds of experiments to establish the effect of Whi3 (RNA abundance by Q-PCR; RNA abundance by RNA-Seq; translating RNA abundance by ribosome profiling; RNA abundance/half-life by comparison of wild-type and gcau mutant RNAs; and measurement of *CLN3* half-life by *GAL-CLN3* shut-off experiments), all giving essentially the same result. In addition, we have repeated the experiments as many times as needed (27 experiments for the comparison of wild-type and gcau mutant RNAs in *WHI3* and *whi3* strains) to drive down the standard error of the mean and obtain very small p-values. In addition, some of our experiments looked at hundreds or thousands of molecules (RNA-Seq, ribosome profiling). Although the effects are small, the p-values we have obtained in each of several very different kinds of experiments are very small, often less than 0.0001, and so we can reliably conclude that Whi3 is reducing abundance of the *CLN3* mRNA.

### Monkey on a Typewriter

What is the point of having an RNA binding protein that slightly destabilizes hundreds or thousands of mRNAs via a frequently-occurring four or five nucleotide motif? It is natural to suppose that such an RNA binding protein might be used to co-ordinately regulate its targets, and this might be true. However, in the case of Whi3 and its targets, it is not easy to ascribe any overall function to the target mRNAs, and furthermore, despite some recent progress suggesting Whi3 could be regulated by phosphorylation [20], [59] (Cai and Futcher, unpublished), there is no clear idea of when and how Whi3 activity is regulated, if indeed it is regulated at all. Furthermore, other investigations of yeast RNA binding proteins and their targets have likewise found an enormous number of targets for many of the RNA binding proteins, with no readily definable overall function for these targets. Here, as a speculation, or perhaps just as a null hypothesis, we would like to state the “monkey on a typewriter” hypothesis, which is the opposite of the “co-ordinate regulation” hypothesis. One can imagine that each mRNA in the cell would have an optimum average half-life, and of course this would vary gene by gene, according to the function of the gene. But what is the mechanism by which one mRNA comes to have a different half-life from another? We suggest that if there is an array of different RNA binding proteins, and some of these increase half-life and some decrease half-life, and if each RNA binding protein recognizes a short motif of four or five nucleotides, then each mRNA can evolve to contain a set of short motifs targeting it to some subset of the RNA binding proteins such that, overall, the mRNA achieves its optimal half-life. In this hypothesis, the clients of a particular mRNA binding protein need have no common function, and need not be co-ordinately regulated. They are independently seeking their optimum half-life. One could say that the RNA binding protein “regulates” its client’s abundance, but over evolutionary time. The fact that the binding motifs are short is consistent with this hypothesis–the shortness of the motif is evidence that the RNA binding protein is not highly specific. (A monkey on a typewriter will only rarely produce “Hamlet”, but will often produce “GCAU”–particularly if the keyboard has only four letters!) The ARE hypothesis–that mRNAs are destabilized by very short AU-rich elements [16]–is a branch of this hypothesis.

## Materials and Methods

### Yeast Strains and Growth

Yeasts were grown in YP medium (1% yeast extract, 2% peptone and 40 mg/L adenine) supplemented with 2% glucose, if not otherwise specified. For transcriptional shut down in *GAL-CLN3* experiments, cells were initially grown to log phase in YP medium with 2% raffinose and 2% galactose, and then glucose was added to a final concentration of 2% to shut off the *GAL* promoter. Cells for microscope imaging were grown in synthetic medium (5% ammonium sulfate, 1.7% yeast nitrogen base) with appropriate amino acids and carbon sources.

Yeast strains are listed in Table 4. Unless otherwise stated, standard genetic techniques were used. Tagging, insertion and deletion was performed by PCR based homologous recombination using cassettes described by Longtine et al. and Janke et al. [57], [60]. To construct the *WHI3x4* strain, pRN200b [1] was linearized with *Bst*EII and incorporated into the genomic locus of *WHI3*. Cell sizes of isolated transformants were measured using a Coulter Counter Z2 and cells with sizes larger than average were selected and Q-PCR was used to quantify the Whi3 transcript levels. A plasmid containing *CLN3* with 12 GCAU sites mutated was obtained from M. Aldea [4]. A *CLN3* length polymorphism with a deletion of base pairs 1582–1650 was made by fusing the 5′ fragment of *CLN3* with the 3′ fragment. The *CLN3* allele with the length polymorphism and the GCAU mutations (“Short-gcau”) was generated by the same PCR reaction but using the GCAU mutant *CLN3* as template. To transplace the wildtype *CLN3* with these mutant *CLN3* alleles, PCR products of these *CLN3* alleles were transformed into a *cln3::CaURA3* strain and cells were selected on 5FOA plates. Then haploid strains carrying different *CLN3* alleles were mated to obtain the diploids with two different versions of *CLN3*. Note that we differ from Aldea and co-workers with regard to the number of GCAU motifs in *CLN3* (12 vs 14) because of a different definition of the 3′ UTR.

**Table 4:** Strains used in this study.

Name	Genotype	Source
BY4741	*MATa his3Δ1 leu2Δ0 met15Δ0 ura3Δ0*	
BY4742	*MATalpha his3Δ1 leu2Δ0 lys2Δ0 ura3Δ0*	
BY4743	*MAT*a/α his3Δ1/his3Δ1 leu2Δ0/leu2Δ0 LYS2/lys2Δ0 met15Δ0/MET15 ura3Δ0/ura3Δ0	
W303a	*MATa,leu2-3,112 trp1-1 can1-100 ura3-1 ade2-1 his3-11,15*	
HWL41	*W303a, WHI3-TAP-KanMX4*	Wang *et al* 2004
CYL4	*W303a, whi3-dRRM-TAP-KanMX4*	this study
CYL21	*BY4741, whi3::URA3*	this study
CYL240	*BY4743, whi3::URA3/whi3::URA3*	this study
CYL81a1	*BY4741, WHI3x4-URA3*	this study
CYL104a	*BY4742, URA3-pGAL-CLN3*	this study
CYL105a	*BY4741, URA3-pGAL-CLN3 whi3::LEU2*	this study
CYL133a	*BY4741,URA3-pGal-CLN3 whi3::LEU2 whi4::KanMX4*	this study
xrn1	*BY4742, xrn1::KanMX4*	Research Genectis deletion set
ccr4	*BY4742, ccr4::KanMX4*	Research Genectis deletion set
CYL178	*BY4743, CLN3/cln3-Short-gcau_mut_*	this study
CYL179	*BY4743*,*cln3-Long-gcau_mut_/cln3-Short-GCAU*	this study
CYL180	*BY4743, CLN3/cln3-Short-GCAU*	this study
CYL181	*BY4743, CLN3/cln3-Short-gcau_mut_* whi3::URA3/whi3::URA3	this study
CYL182	*BY4743*, *cln3-Long-gcau_mut_/cln3-Short-GCAU whi3::URA3/whi3::URA3*	this study
CYL183a	*BY4743, CLN3/cln3-Short-GCAU whi3::URA3/whi3::URA3*	this study
WHI3-GFP	*BY4741, WHI3-GFP-HIS3*	yeast GFP collection
CYL99a	*BY4741, whi3-dQ-GFP-HIS3*	this study
CYL156a	*BY4741, whi3-dRRM-GFP-hphNT1*	this study
CYL169a	*BY4741, whi3-dQdRRM-GFP-hphNT1*	this study
CYL76a	*BY4742, CLN3-MS2L*	this study
CYL82a	*BY4742, CLN3-MS2L whi3::URA3*	this study
CYL93a	*BY4742, CLN3-MS2L whi3::URA3 whi4::KanMX4*	this study
CYL194a	*BY4742, PGK1-MS2L*	this study
CYL130c	*BY4741, whi3::LEU2 xrn1::KanMX4*	this study

### Plasmids

Plasmids used in this study are listed in Table 5.

**Table 5:** Plasmids used in this study.

Name	character	source
pRP1155	Dcp2-RFP, *CEN*, *LEU2*	Teixeira, *et al* 2005
pRP1574	Edc3-mCherry, *CEN*,*URA3*	Buchan, *et al* 2008
pRP1661	Pub1-mCherry, *CEN*,*URA3*	Buchan, *et al* 2009
YCplacIII-MS2_GFP	NLS-MS2CP-GFP, *CEN*, LEU2	Gu, *et al* 2004
pRP1155-LYS2	Dcp2-RFP, *CEN*, *LYS2*	this study
pSH47	*pGAL-CRE*, *CEN*, *URA3*	Haim, *et al* 2007
pLoxHIS5MS2L	for integration of MS2L-loxP-Sphis5^+^-loxP	Haim, *et al* 2007
pRN200b	pRS306 with insertion fragment of DNA containig*WHI3*, for construct *WHI3x4*	Nash, *et al* 2001
pCYC2072	*cln3-GCAU^mut^*, *CEN*, *URA3*	Colomina, *et al* 2008

### RIP-chip and Microarray

8L of yeast cells carrying Whi3 tagged with the TAP tag were grown in YP to log phase. Cells were harvested, resuspended in cold lysis buffer (10 mM Hepes-Na, pH 7.5, 10 mM KCl, 1.5 mM MgCl_2_, 0.5 mM DTT and protease inhibitors) and lysed by passing through a French press twice. KCl and Triton were added to the extract to a final concentration of 200 mM KCl and 1% Triton. After centrifugation at 4000 rpm for 5 min and 15000 rpm for 15 min at 4°C, aliquots of the supernatants were saved for assays of input protein and RNA, and the rest was passed through columns filled with 100 µl IgG Sepharose 6 Fast Flow (GE Healthcare) (The protein A moiety of the TAP tag binds to IgG.). After three washes with IPP150 (10 mM Tris-Cl, pH 8, 150 mM NaCl, 0.1% Triton), the IgG beads were resuspended in 600 µl TES (10 mM Tris-HCl, pH 8, 10 mM EDTA, pH 8, 0.5% SDS). To isolate the RNA associated with the beads, an equal volume of acid phenol was added, vortexed and incubated at 65°C for 15 min. After two rounds of acid phenol extraction, the isolated RNA was ethanol precipitated from the aqueous phase for labeling.

### Microarray

The microarrays for RNA-IP (RIP-chip) were performed on homemade arrays. Manufacture of these arrays was described in Spellman et al. [61]. 20–25 µg input RNA and all of the immunoprecipitated RNA was used for labeling by an aminoallyl labeling method adapted from The Institute for Genomic Research, Standard Operating Procedure SOP #M004. Briefly, the RNA was reverse transcribed with SuperScript®II (invitrogen) and Oligo-dT, 2 mM dNTPs and 0.3 mM amino-allyl-dUTP (Ambion) was added for incorporation into the cDNA. The labeled cDNA was purified and coupled with the appropriate NHS-ester Cy-dye (Amersham). All the labeled cDNAs from immunoprecipitated RNA were hybridized against a total RNA normalization control containing 50 pmol of incorporated dye. Arrays were scanned using a GenePix 4000B (Axon Instruments) controlled by GenePix Pro 5.1 software. Images were quantified using GenePix Pro. After background subtraction and loess normalization, the log ratios from multiple independent spots with the same probe were averaged. To obtain the list of Whi3 targets, a net log ratio was calculated by subtract the ratio of the control experiment from that of the Whi3 IP experiment. The whole experiment was done independently twice, and the net log ratios were averaged. RNAs with net log ratios that were 1.65 standard deviations above the mean were defined as Whi3 targets. We chose to make a relatively inclusive list (i.e. requiring only 1.65 standard deviations above the mean) in part because it gave a list of roughly the same length as the previous list of Colomina et al. [4] (262 genes on our list; 326 genes for Colomina et al.).

The expression microarrays for the *whi3* deletion and *WHI3x4* strains were performed on Yeast (V2) Gene Expression Microarrays, 8×15 K (Agilent) following the Agilent labeling protocol “Two-Color Microarray-Based Gene Expression Analysis (Quick Amp Labeling)” with Tecan HS Pro Hybridization. In the expression arrays, RNA from WT cells under the same growth conditions was used as the reference. Data were retrieved and loess normalized by Feature Extraction software (Agilent). Flagged spots were removed before subsequent analysis.

Microarray data can be found in the NCBI’s GEO database under accession number GSE51784 (RIP-chip) and GSE51165 (expression array).

### Total RNA Isolation and Quantification of mRNA Level by Q-PCR

Total RNA was isolated using a RiboPure Yeast Kit (Ambion) following the provided protocol. 10 µg of RNA treated with Turbo DNase (Ambion) was used for reverse transcription (Superscript® III, invitrogen) using Oligo-dT. The amount of mRNA was quantified by Q-PCR. The Q-PCR reaction was performed on an Eppendorf Mastercycler® RealPlex2 (Eppendorf) in a standard Q-PCR program at 10 µl total volumes, using FastStart SYBR Green Master (Roche). All the mRNA levels were normalized against *ACT1* mRNA.

### PARS Analysis

RNA sequences consisting of 50 nucleotides flanking GCAU, UGCAU or AUUUUA from RNAs were retrieved, categorized into sequences from Whi3 targets or non-targets based on our list of 262 mRNAs, and aligned with the motif centered in the middle. The PARS score (cut frequency by S1 or V1) [19] was averaged for each aligned position separately for Whi3 targets and non-targets. The averaged score was compared between Whi3 targets and non-targets. The same analysis was done for CGUA and AUCG as negative controls.

### Ligation-Mediated Poly(A) Test (LM-PAT)

Total RNA was prepared as described above. Ligation and reverse transcription was performed according to the protocol described by Salles and Strickland [38]. cDNA was PCR amplified and detected via 10% polyacrylamide gel electrophoresis and ethidium bromide staining.

### GCAU Analysis

Gene sequences were obtained from SGD. 5′UTRs and 3′UTRs were defined according to the RNA-Seq data from Nagalakshmi et al. [62]. For genes for which UTR information was not available, 200 nucleotides upstream or downstream of the ORF were used as the 5′UTR or 3′UTR respectively. If not specifically mentioned, GCAU density was calculated as the number of GCAUs per kb in the gene including both the 5′UTR and the 3′UTR. To calculate local maximum GCAU frequency, the maximum number of GCAUs in a sliding window of 300 nucleotides was recorded for each gene.

### Long/Short *CLN3* GCAU Experiment

Semi-quantitative PCR was performed using a pair of oligos flanking the deleted region in the *CLN3* length polymorphism to differentiate the long and short *CLN3* mRNA species. Different numbers of PCR amplification cycles were tested to make sure the amplification reaction was in the linear range. PCR products were separated by gel electrophoresis and stained with ethidium bromide. The intensity of the two bands was quantified by ImageJ.

### Ribosome Profiling and RNA-Seq

1 liter of homozygous diploid WT or *whi3* cells was grown in YPD to log phase. The culture was mixed with ice and cycloheximide at the same time (final CHX concentration was 100 µg/ml). Cells were washed once with ice cold water with 100 µg/ml cycloheximide. An ARTseq™ Ribosome Profiling Kit was used to generate a ribosome footprint library and total RNA library. The given protocol was followed strictly except that an additional rRNA removal step using biotinylated oligos was performed before the end repair step.

### Sequence Analysis for RNA-Seq and Ribosome Profiling

Sequence data from an Illumina HiSeq were initially trimmed and low quality reads were filtered using the FASTX-toolkit. The first 26 nucleotides of the sequencing reads were used for alignment using Bowtie2 with the end-to-end alignment method against S288C reference genome R64. Unaligned reads were recycled for additional alignment using coding region sequence as reference to retrieve exon junction reads. The position of the 5′ end in the read was used to assign reads to genomic features [43]. Reads mapped to the rDNA were discarded for following analysis. Reads per kilobase per million reads (RPKM) was calculated to quantify mRNA and ribosomal footprints. Briefly, after removal of genes with less than 7 reads, the count of reads for each gene was normalized by its length (in kb) and the total number of reads (in million). RNA-seq and ribosome profiling data can be found in the NCBI’s GEO database under accession number GSE51164.

### Microscopy

For glucose starvation experiments, log phase cells were washed, resuspended with the same medium but lacking glucose, and incubated for 30 min before imaging. For heat shock experiments, log phase cells were collected by centrifugation, resuspended in the same medium prewarmed to 46°C and incubated at 46°C for 10 min before imaging. To prepare cells for microscopy, concentrated cell culture was mounted onto prewarmed agarose plates and pictures were taken immediately after the glucose starvation or the heatshock treatment. Observations were made using either a Zeiss Axioplan2 microscope with a Zeiss mRM Axiocam (Carl Zeiss) or a Zeiss Observer.Z1 microscope with an attached Orca II ERG camera (Hamamatsu). Zeiss Axiovision 4.8 was used to acquire images. Z-stack images were made by a Z-series compilation of 4–7 images. P-body markers and stress granule marker were all ectopically expressed and the plasmids used are listed in Table 5. 12 copies of the MS2 loop were integrated between the ORF and 3′UTR by the method developed by Haim et al. [55]. To visualize RNA localization, cells tagged with MS2 loops were transformed a plasmid containing MS2CP-GFP which binds specifically to the MS2 loop of RNA [56].

## Supporting Information

Table S1 **Whi3 RIP-chip.** This spreadsheet contains separate tabs for the full RIP-chip results (Whi3_RIP-chip) and the RIP-chip results for the 262 mRNAs identified as Whi3 targets in this study (Whi3_targets). In both datasets, net_IP_ratio is the averaged net IP ratio as described in the paper. The rank of the net_IP_ratio is also listed.
(XLSX)

Table S2 **Joint list of Whi3 targets.** This table contains a set of 111 Whi3 target mRNAs common between this study and the study by Colomina et al. The net_IP_ratio and rank are the averaged net IP ratio and the rank of the net IP ratio from this study. IP_col and rank_col are the IP ratio and the rank of the IP ratio from Colomina et al. A description from SGD for each gene is also given.
(XLSX)

Table S3 **Microarray of** ***whi3*** **and** ***WHI4X4*** **.** This table contains the processed data from the microarray experiment on the *whi3* and the *WHI3X4* strain. whi3_YPD and WHI3X4_YPD are the log2 ratios of mutant over WT for *whi3* and *WHI3X4* cells grown in YP glucose medium. whi3_YPE and WHI3X4_YPE are the log2 ratios of mutant over WT for *whi3* and *WHI3X4* cells grown in YP ethanol medium.
(XLSX)

## References

[1] Nash RS, Volpe T, Futcher B (2001) Isolation and characterization of WHI3, a size-control gene of Saccharomyces cerevisiae. Genetics 157: 1469–1480. 11290704 10.1093/genetics/157.4.1469 PMC1461599

[2] Gari E, Volpe T, Wang H, Gallego C, Futcher B, et al (2001) Whi3 binds the mRNA of the G1 cyclin CLN3 to modulate cell fate in budding yeast. Genes Dev 15: 2803–2808. 11691832 10.1101/gad.203501 PMC312816

[3] Nash R, Tokiwa G, Anand S, Erickson K, Futcher AB (1988) The WHI1+ gene of Saccharomyces cerevisiae tethers cell division to cell size and is a cyclin homolog. EMBO J 7: 4335–4346. 2907481 10.1002/j.1460-2075.1988.tb03332.x PMC455150

[4] Colomina N, Ferrezuelo F, Wang H, Aldea M, Gari E (2008) Whi3, a developmental regulator of budding yeast, binds a large set of mRNAs functionally related to the endoplasmic reticulum. J Biol Chem 283: 28670–28679. 18667435 10.1074/jbc.M804604200 PMC2661415

[5] Wang H, Gari E, Verges E, Gallego C, Aldea M (2004) Recruitment of Cdc28 by Whi3 restricts nuclear accumulation of the G1 cyclin-Cdk complex to late G1. EMBO J 23: 180–190. 14685274 10.1038/sj.emboj.7600022 PMC1271660

[6] Lee C, Zhang H, Baker AE, Occhipinti P, Borsuk ME, et al (2013) Protein aggregation behavior regulates cyclin transcript localization and cell-cycle control. Dev Cell 25: 572–584. 23769973 10.1016/j.devcel.2013.05.007 PMC4113091

[7] Verges E, Colomina N, Gari E, Gallego C, Aldea M (2007) Cyclin Cln3 is retained at the ER and released by the J chaperone Ydj1 in late G1 to trigger cell cycle entry. Mol Cell 26: 649–662. 17560371 10.1016/j.molcel.2007.04.023

[8] Miller ME, Cross FR (2001) Mechanisms controlling subcellular localization of the G(1) cyclins Cln2p and Cln3p in budding yeast. Mol Cell Biol 21: 6292–6311. 11509671 10.1128/MCB.21.18.6292-6311.2001 PMC87357

[9] Miller ME, Cross FR (2000) Distinct subcellular localization patterns contribute to functional specificity of the Cln2 and Cln3 cyclins of Saccharomyces cerevisiae. Mol Cell Biol 20: 542–555. 10611233 10.1128/mcb.20.2.542-555.2000 PMC85127

[10] Lockshon D, Surface LE, Kerr EO, Kaeberlein M, Kennedy BK (2007) The sensitivity of yeast mutants to oleic acid implicates the peroxisome and other processes in membrane function. Genetics 175: 77–91. 17151231 10.1534/genetics.106.064428 PMC1774995

[11] Mizunuma M, Tsubakiyama R, Ogawa T, Shitamukai A, Kobayashi Y, et al (2013) Ras/cAMP-dependent protein kinase (PKA) regulates multiple aspects of cellular events by phosphorylating the Whi3 cell cycle regulator in budding yeast. J Biol Chem 288: 10558–10566. 23471970 10.1074/jbc.M112.402214 PMC3624437

[12] Budovskaya YV, Stephan JS, Deminoff SJ, Herman PK (2005) An evolutionary proteomics approach identifies substrates of the cAMP-dependent protein kinase. Proc Natl Acad Sci U S A 102: 13933–13938. 16172400 10.1073/pnas.0501046102 PMC1236527

[13] Sengupta DJ, Wickens M, Fields S (1999) Identification of RNAs that bind to a specific protein using the yeast three-hybrid system. RNA 5: 596–601. 10199575 10.1017/s1355838299002113 PMC1369785

[14] Riordan DP, Herschlag D, Brown PO (2011) Identification of RNA recognition elements in the Saccharomyces cerevisiae transcriptome. Nucleic Acids Res 39: 1501–1509. 20959291 10.1093/nar/gkq920 PMC3045596

[15] Elemento O, Slonim N, Tavazoie S (2007) A universal framework for regulatory element discovery across all genomes and data types. Mol Cell 28: 337–350. 17964271 10.1016/j.molcel.2007.09.027 PMC2900317

[16] Gillis P, Malter JS (1991) The adenosine-uridine binding factor recognizes the AU-rich elements of cytokine, lymphokine, and oncogene mRNAs. J Biol Chem 266: 3172–3177. 1993689

[17] Malter JS, McCrory WA, Wilson M, Gillis P (1990) Adenosine-uridine binding factor requires metals for binding to granulocyte-macrophage colony-stimulating factor mRNA. Enzyme 44: 203–213. 2133652 10.1159/000468758

[18] Vasudevan S, Peltz SW (2001) Regulated ARE-mediated mRNA decay in Saccharomyces cerevisiae. Mol Cell 7: 1191–1200. 11430822 10.1016/s1097-2765(01)00279-9

[19] Kertesz M, Wan Y, Mazor E, Rinn JL, Nutter RC, et al (2010) Genome-wide measurement of RNA secondary structure in yeast. Nature 467: 103–107. 20811459 10.1038/nature09322 PMC3847670

[20] Malcher M, Schladebeck S, Mosch HU (2011) The Yak1 protein kinase lies at the center of a regulatory cascade affecting adhesive growth and stress resistance in Saccharomyces cerevisiae. Genetics 187: 717–730. 21149646 10.1534/genetics.110.125708 PMC3063667

[21] Laabs TL, Markwardt DD, Slattery MG, Newcomb LL, Stillman DJ, et al (2003) ACE2 is required for daughter cell-specific G1 delay in Saccharomyces cerevisiae. Proc Natl Acad Sci U S A 100: 10275–10280. 12937340 10.1073/pnas.1833999100 PMC193551

[22] Tyers M, Tokiwa G, Nash R, Futcher B (1992) The Cln3-Cdc28 kinase complex of S. cerevisiae is regulated by proteolysis and phosphorylation. EMBO J 11: 1773–1784. 1316273 10.1002/j.1460-2075.1992.tb05229.x PMC556635

[23] Tyers M, Fitch I, Tokiwa G, Dahmann C, Nash R, et al (1991) Characterization of G1 and mitotic cyclins of budding yeast. Cold Spring Harb Symp Quant Biol 56: 21–32. 1840252 10.1101/sqb.1991.056.01.005

[24] Schneider BL, Zhang J, Markwardt J, Tokiwa G, Volpe T, et al (2004) Growth rate and cell size modulate the synthesis of, and requirement for, G1-phase cyclins at start. Mol Cell Biol 24: 10802–10813. 15572683 10.1128/MCB.24.24.10802-10813.2004 PMC533974

[25] Hall DD, Markwardt DD, Parviz F, Heideman W (1998) Regulation of the Cln3-Cdc28 kinase by cAMP in Saccharomyces cerevisiae. EMBO J 17: 4370–4378. 9687505 10.1093/emboj/17.15.4370 PMC1170770

[26] Polymenis M, Schmidt EV (1997) Coupling of cell division to cell growth by translational control of the G1 cyclin CLN3 in yeast. Genes Dev 11: 2522–2531. 9334317 10.1101/gad.11.19.2522 PMC316559

[27] Gallego C, Gari E, Colomina N, Herrero E, Aldea M (1997) The Cln3 cyclin is down-regulated by translational repression and degradation during the G1 arrest caused by nitrogen deprivation in budding yeast. EMBO J 16: 7196–7206. 9384596 10.1093/emboj/16.23.7196 PMC1170320

[28] Tarassov K, Messier V, Landry CR, Radinovic S, Serna Molina MM, et al (2008) An in vivo map of the yeast protein interactome. Science 320: 1465–1470. 18467557 10.1126/science.1153878

[29] Collart MA, Panasenko OO (2012) The Ccr4–not complex. Gene 492: 42–53. 22027279 10.1016/j.gene.2011.09.033

[30] Wahle E, Winkler GS (2013) RNA decay machines: deadenylation by the Ccr4-not and Pan2-Pan3 complexes. Biochim Biophys Acta 1829: 561–570. 23337855 10.1016/j.bbagrm.2013.01.003

[31] Wiederhold K, Passmore LA (2010) Cytoplasmic deadenylation: regulation of mRNA fate. Biochem Soc Trans 38: 1531–1536. 21118121 10.1042/BST0381531 PMC3890232

[32] Tucker M, Staples RR, Valencia-Sanchez MA, Muhlrad D, Parker R (2002) Ccr4p is the catalytic subunit of a Ccr4p/Pop2p/Notp mRNA deadenylase complex in Saccharomyces cerevisiae. EMBO J 21: 1427–1436. 11889048 10.1093/emboj/21.6.1427 PMC125913

[33] Muhlrad D, Decker CJ, Parker R (1995) Turnover mechanisms of the stable yeast PGK1 mRNA. Mol Cell Biol 15: 2145–2156. 7891709 10.1128/mcb.15.4.2145 PMC230442

[34] Muhlrad D, Decker CJ, Parker R (1994) Deadenylation of the unstable mRNA encoded by the yeast MFA2 gene leads to decapping followed by 5′–>3′ digestion of the transcript. Genes Dev 8: 855–866. 7926773 10.1101/gad.8.7.855

[35] Tucker M, Valencia-Sanchez MA, Staples RR, Chen J, Denis CL, et al (2001) The transcription factor associated Ccr4 and Caf1 proteins are components of the major cytoplasmic mRNA deadenylase in Saccharomyces cerevisiae. Cell 104: 377–386. 11239395 10.1016/s0092-8674(01)00225-2

[36] Tucker M, Parker R (2000) Mechanisms and control of mRNA decapping in Saccharomyces cerevisiae. Annu Rev Biochem 69: 571–595. 10966469 10.1146/annurev.biochem.69.1.571

[37] Salles FJ, Strickland S (1999) Analysis of poly(A) tail lengths by PCR: the PAT assay. Methods Mol Biol 118: 441–448. 10549542 10.1385/1-59259-676-2:441

[38] Salles FJ, Strickland S (1995) Rapid and sensitive analysis of mRNA polyadenylation states by PCR. PCR Methods Appl 4: 317–321. 7580923 10.1101/gr.4.6.317

[39] Hsu CL, Stevens A (1993) Yeast cells lacking 5′–>3′ exoribonuclease 1 contain mRNA species that are poly(A) deficient and partially lack the 5′ cap structure. Mol Cell Biol 13: 4826–4835. 8336719 10.1128/mcb.13.8.4826-4835.1993 PMC360109

[40] Larimer FW, Stevens A (1990) Disruption of the gene XRN1, coding for a 5′–-3′ exoribonuclease, restricts yeast cell growth. Gene 95: 85–90. 1979303 10.1016/0378-1119(90)90417-p

[41] Ingolia NT, Brar GA, Rouskin S, McGeachy AM, Weissman JS (2012) The ribosome profiling strategy for monitoring translation in vivo by deep sequencing of ribosome-protected mRNA fragments. Nat Protoc 7: 1534–1550. 22836135 10.1038/nprot.2012.086 PMC3535016

[42] Brar GA, Yassour M, Friedman N, Regev A, Ingolia NT, et al (2012) High-resolution view of the yeast meiotic program revealed by ribosome profiling. Science 335: 552–557. 22194413 10.1126/science.1215110 PMC3414261

[43] Ingolia NT, Ghaemmaghami S, Newman JR, Weissman JS (2009) Genome-wide analysis in vivo of translation with nucleotide resolution using ribosome profiling. Science 324: 218–223. 19213877 10.1126/science.1168978 PMC2746483

[44] Beckham C, Hilliker A, Cziko AM, Noueiry A, Ramaswami M, et al (2008) The DEAD-box RNA helicase Ded1p affects and accumulates in Saccharomyces cerevisiae P-bodies. Mol Biol Cell 19: 984–993. 18162578 10.1091/mbc.E07-09-0954 PMC2262982

[45] Nissan T, Parker R (2008) Analyzing P-bodies in Saccharomyces cerevisiae. Methods Enzymol 448: 507–520. 19111192 10.1016/S0076-6879(08)02625-6 PMC2693489

[46] Dunckley T, Parker R (1999) The DCP2 protein is required for mRNA decapping in Saccharomyces cerevisiae and contains a functional MutT motif. EMBO J 18: 5411–5422. 10508173 10.1093/emboj/18.19.5411 PMC1171610

[47] Teixeira D, Sheth U, Valencia-Sanchez MA, Brengues M, Parker R (2005) Processing bodies require RNA for assembly and contain nontranslating mRNAs. RNA 11: 371–382. 15703442 10.1261/rna.7258505 PMC1370727

[48] Kshirsagar M, Parker R (2004) Identification of Edc3p as an enhancer of mRNA decapping in Saccharomyces cerevisiae. Genetics 166: 729–739. 15020463 10.1093/genetics/166.2.729 PMC1470743

[49] Buchan JR, Parker R (2009) Eukaryotic stress granules: the ins and outs of translation. Mol Cell 36: 932–941. 20064460 10.1016/j.molcel.2009.11.020 PMC2813218

[50] Shah KH, Zhang B, Ramachandran V, Herman PK (2013) Processing body and stress granule assembly occur by independent and differentially regulated pathways in Saccharomyces cerevisiae. Genetics 193: 109–123. 23105015 10.1534/genetics.112.146993 PMC3527240

[51] Buchan JR, Muhlrad D, Parker R (2008) P bodies promote stress granule assembly in Saccharomyces cerevisiae. J Cell Biol 183: 441–455. 18981231 10.1083/jcb.200807043 PMC2575786

[52] Swisher KD, Parker R (2010) Localization to, and effects of Pbp1, Pbp4, Lsm12, Dhh1, and Pab1 on stress granules in Saccharomyces cerevisiae. PLoS One 5: e10006. 20368989 10.1371/journal.pone.0010006 PMC2848848

[53] Santoso A, Chien P, Osherovich LZ, Weissman JS (2000) Molecular basis of a yeast prion species barrier. Cell 100: 277–288. 10660050 10.1016/s0092-8674(00)81565-2

[54] Reijns MA, Alexander RD, Spiller MP, Beggs JD (2008) A role for Q/N-rich aggregation-prone regions in P-body localization. J Cell Sci 121: 2463–2472. 18611963 10.1242/jcs.024976 PMC2680509

[55] Haim L, Zipor G, Aronov S, Gerst JE (2007) A genomic integration method to visualize localization of endogenous mRNAs in living yeast. Nat Methods 4: 409–412. 17417645 10.1038/nmeth1040

[56] Gu W, Deng Y, Zenklusen D, Singer RH (2004) A new yeast PUF family protein, Puf6p, represses ASH1 mRNA translation and is required for its localization. Genes Dev 18: 1452–1465. 15198983 10.1101/gad.1189004 PMC423195

[57] Longtine MS, McKenzie A 3rd, Demarini DJ, Shah NG, Wach A, et al (1998) Additional modules for versatile and economical PCR-based gene deletion and modification in Saccharomyces cerevisiae. Yeast 14: 953–961. 9717241 10.1002/(SICI)1097-0061(199807)14:10<953::AID-YEA293>3.0.CO;2-U

[58] Chen HM, Futcher B, Leatherwood J (2011) The fission yeast RNA binding protein Mmi1 regulates meiotic genes by controlling intron specific splicing and polyadenylation coupled RNA turnover. PLoS One 6: e26804. 22046364 10.1371/journal.pone.0026804 PMC3203177

[59] Mizunuma M, Tsubakiyama R, Ogawa T, Shitamukai A, Kobayashi Y, et al (2013) Ras/cAMP-dependent protein kinase (PKA) regulates multiple aspects of cellular events by phosphorylating the Whi3 cell cycle regulator in budding yeast. J Biol Chem 288: 10558–10566. 23471970 10.1074/jbc.M112.402214 PMC3624437

[60] Janke C, Magiera MM, Rathfelder N, Taxis C, Reber S, et al (2004) A versatile toolbox for PCR-based tagging of yeast genes: new fluorescent proteins, more markers and promoter substitution cassettes. Yeast 21: 947–962. 15334558 10.1002/yea.1142

[61] Spellman PT, Sherlock G, Zhang MQ, Iyer VR, Anders K, et al (1998) Comprehensive identification of cell cycle-regulated genes of the yeast Saccharomyces cerevisiae by microarray hybridization. Mol Biol Cell 9: 3273–3297. 9843569 10.1091/mbc.9.12.3273 PMC25624

[62] Nagalakshmi U, Wang Z, Waern K, Shou C, Raha D, et al (2008) The transcriptional landscape of the yeast genome defined by RNA sequencing. Science 320: 1344–1349. 18451266 10.1126/science.1158441 PMC2951732

